# Targeting collagen XVIII improves the efficiency of ErbB inhibitors in breast cancer models

**DOI:** 10.1172/JCI159181

**Published:** 2023-09-15

**Authors:** Raman Devarajan, Valerio Izzi, Hellevi Peltoketo, Gunilla Rask, Saila Kauppila, Marja-Riitta Väisänen, Heli Ruotsalainen, Guillermo Martínez-Nieto, Sanna-Maria Karppinen, Timo Väisänen, Inderjeet Kaur, Jussi Koivunen, Takako Sasaki, Robert Winqvist, Aki Manninen, Fredrik Wärnberg, Malin Sund, Taina Pihlajaniemi, Ritva Heljasvaara

**Affiliations:** 1 Oulu Center for Cell-Matrix Research, Faculty of Biochemistry and Molecular Medicine,; 2 Laboratory of Cancer Genetics and Tumor Biology, Cancer and Translational Medicine Research Unit,; 3 Biocenter Oulu, and; 4 Research Unit of Biomedicine, University of Oulu, Oulu, Finland.; 5 Finnish Cancer Research Institute, Helsinki, Finland.; 6 Department of Medical Biosciences/Pathology, Umeå University, Umeå, Sweden.; 7 Department of Pathology, Oulu University Hospital and University of Oulu, Oulu, Finland.; 8 Northern Finland Laboratory Centre, NordLab, Oulu, Finland.; 9 Department of Pathology, Kainuu Central Hospital, Kajaani, Finland.; 10 Department of Medical Oncology and Radiotherapy and Medical Research Center, Oulu University Hospital and University of Oulu, Oulu, Finland.; 11 Department of Pharmacology, Faculty of Medicine, Oita University, Oita, Japan.; 12 Disease Networks Research Unit, Faculty of Biochemistry and Molecular Medicine, University of Oulu, Oulu, Finland.; 13 Department of Surgery, Institute of Clinical Sciences, Sahlgrenska Academy at the University of Gothenburg, Gothenburg, Sweden.; 14 Department of Surgery and Perioperative Sciences/Surgery, Umeå University, Umeå, Sweden.; 15 Department of Surgery, University of Helsinki and Helsinki University Hospital, Helsinki, Finland.

**Keywords:** Oncology, Breast cancer, Collagens, Growth factors

## Abstract

The tumor extracellular matrix (ECM) critically regulates cancer progression and treatment response. Expression of the basement membrane component collagen XVIII (ColXVIII) is induced in solid tumors, but its involvement in tumorigenesis has remained elusive. We show here that ColXVIII was markedly upregulated in human breast cancer (BC) and was closely associated with a poor prognosis in high-grade BCs. We discovered a role for ColXVIII as a modulator of epidermal growth factor receptor tyrosine kinase (ErbB) signaling and show that it forms a complex with ErbB1 and -2 (also known as EGFR and human epidermal growth factor receptor 2 [HER2]) and α6-integrin to promote cancer cell proliferation in a pathway involving its N-terminal portion and the MAPK/ERK1/2 and PI3K/AKT cascades. Studies using *Col18a1* mouse models crossed with the mouse mammary tumor virus–polyoma virus middle T antigen (MMTV-PyMT) mammary carcinogenesis model showed that ColXVIII promoted BC growth and metastasis in a tumor cell–autonomous manner. Moreover, the number of mammary cancer stem cells was significantly reduced in the MMTV-PyMT and human cell models upon ColXVIII inhibition. Finally, ablation of ColXVIII substantially improved the efficacy of ErbB-targeting therapies in both preclinical models. In summary, ColXVIII was found to sustain the stemness properties of BC cells and tumor progression and metastasis through ErbB signaling, suggesting that targeting ColXVIII in the tumor milieu may have important therapeutic potential.

## Graphical abstract

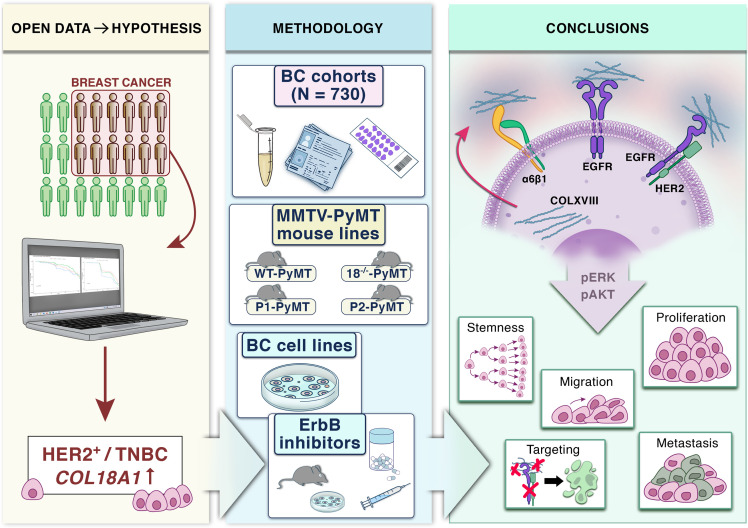

## Introduction

Breast cancer (BC) is the most common cancer among women, with over 2 million new cases diagnosed in 2020, and accounts for 25% of all female cancers (1). Treatment options depend on the type and course of the disease, hormone and human epidermal growth factor receptor 2 (HER2) status, mutations, proliferation index, and differentiation score (2). Hence, patients with BC are treated with different combinations of surgery, radiation, chemotherapy, and endocrine therapy as well as with targeted immunotherapies or small-molecule inhibitor (SMI) therapies.

Clinical studies show that epidermal growth factor receptor tyrosine kinases (EGFR, also known as ErbB) have important roles in the development and progression of BC (3). In BC, HER2 is clinically the most relevant of the 4 ErbBs, as it is amplified or overexpressed in 20%–30% of BC cases and is associated with an aggressive cancer type and poor prognosis (4). The development of HER2-targeted therapies, the mAb trastuzumab in particular, has improved the survival of patients with HER2^+^ tumors (4). EGFR (also known ErbB1 or HER1) is overexpressed in 15%–30% of all BCs, most frequently in the aggressive triple-negative BCs (TNBCs) and inflammatory BCs, and it is also associated with large tumor size, poor differentiation, and poor clinical outcomes for these tumor types (5).

A major challenge in cancer treatment is intrinsic or acquired drug resistance, which is responsible for most of the relapses that occur after an initially favorable response (6). For example, approximately 70% of advanced HER2-type BCs develop resistance to trastuzumab and progress to metastatic disease. Many patients also become resistant to lapatinib, a dual SMI of HER2 and EGFR (7, 8). In addition, residual disease in the breast or lymph nodes after neoadjuvant chemotherapy confers a high risk of recurrence in TNBC (9, 10).

While genetic alterations in cells predispose individuals to, initiate, and drive malignancy, cancer progression is enabled by a dysregulated tumor microenvironment (TME), comprising different types of stromal cells and the extracellular matrix (ECM) (11, 12). Both tumor and stromal cells actively produce ECM proteins and ECM-modifying enzymes to remodel the TME, which then promotes the growth of cancer cells and their invasion into the surrounding tissue and beyond (12). Moreover, biological and mechanical cues from the ECM support the acquisition of cancer stem cell (CSC) properties by somatic tumor cells, thus favoring tumor growth, development of drug resistances, and, eventually, disease relapse (13, 14). We showed that the expression of ECM components in cancers is precisely regulated by specific oncogenic drivers and transcription factors and correlates with the patient’s prognosis (15). This study and many others, including our recent studies (16–18), highlight the utility of ECM molecules as prognostic and predictive biomarkers and unveil new therapeutic possibilities for inhibiting cancer progression, metastasis, and drug resistances by targeting the ECM (12–14).

Collagen XVIII (ColXVIII) is a ubiquitous component of epithelial and endothelial basement membranes (BMs) (19). It is a structurally complex and functionally versatile molecule with characterized roles in the eye, nervous system, and adipose tissue, for example. ColXVIII exists in 3 isoforms — short, medium, and long — which differ in their N-terminal noncollagenous (NC) domain structure (Supplemental Figure 1A; supplemental material available online with this article; https://doi.org/10.1172/JCI159181DS1), tissue specificity, and functions. All isoforms contain an antiangiogenic endostatin (ES) domain in their C-terminal NC1 portion and a laminin-G/thrombospondin-1–like (TSP-1) domain in their N-terminal NC11 portion. The long ColXVIII has 2 additional domains in the N-terminus, a mucin-like domain (MUCL-C18) and a Wnt-binding Frizzled-like domain (FZ-C18), which is spliced out of the medium ColXVIII (19, 20). In several neoplasms, including lung, prostate, and gastric cancers, both ColXVIII overexpression in tumor tissues and high plasma ES levels have been associated with disease progression and poor prognosis rather than with tumor repression by ES (20). However, the mechanisms by which ColXVIII promotes tumor growth and progression are still unclear.

We set out here to investigate the role and molecular mechanisms of ColXVIII in BC using genetic mouse tumor models and human BC cell models. In addition, we assessed the translational value of ColXVIII by correlating its expression with the clinicopathological features in a patient cohort with over 600 BC specimens and in public databases, and by conducting drug tests in cell and mouse models. Our studies revealed a mechanism for ColXVIII in the regulation of ErbB signaling in BC that leads to tumor promotion and demonstrated marked upregulation of ColXVIII in high-grade BCs that was associated with a poor clinical outcome. Our preclinical assays showed that ColXVIII targeting has promising therapeutic potential in the treatment of BC.

## Results

### ColXVIII expression in human BCs.

**Figure 1:**
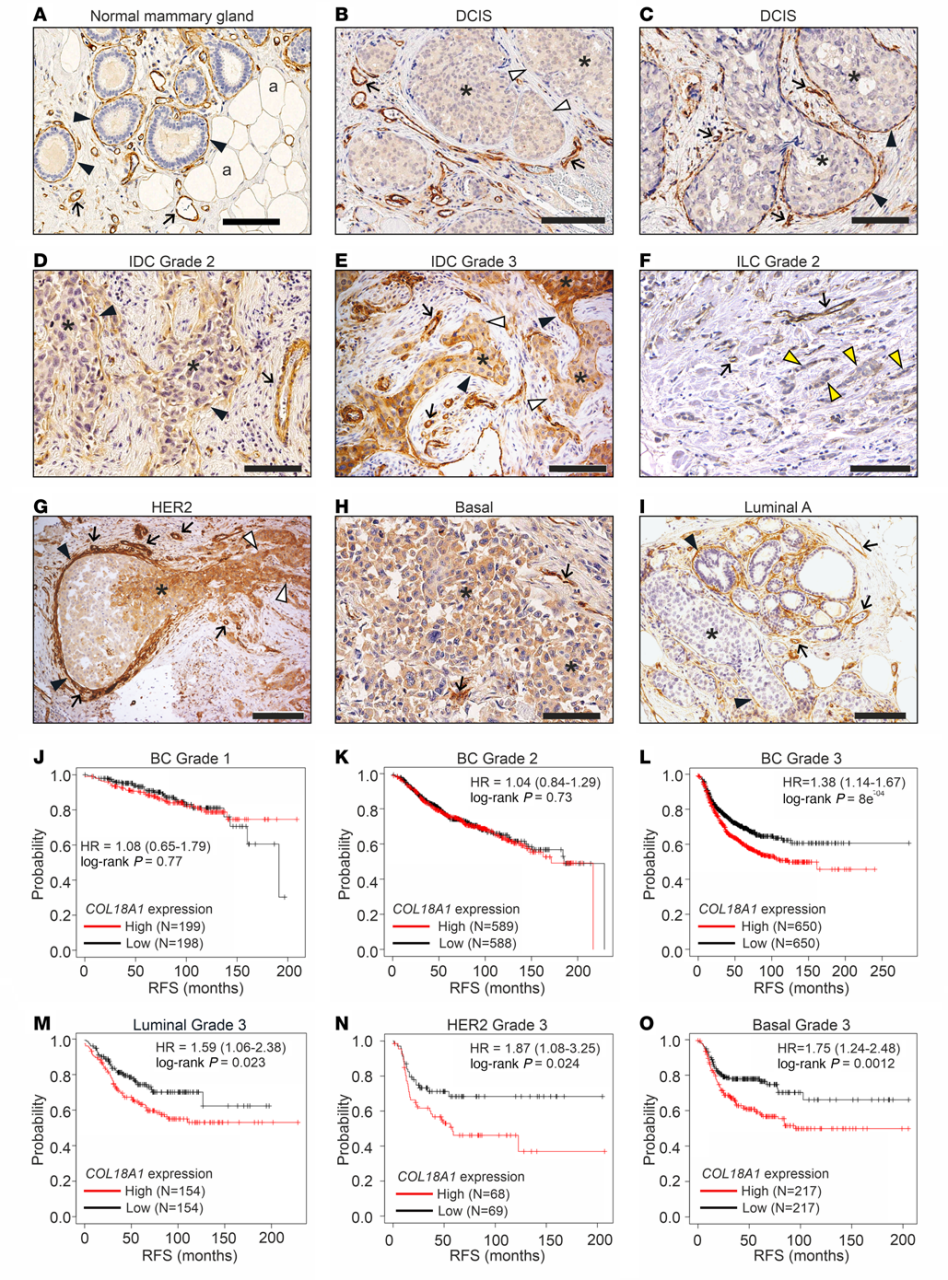
High ColXVIII expression is associated with poor prognosis for human BC. (**A**–**I**). Representative images of ColXVIII expression and localization in (**A**) normal breast tissue, (**B** and **C**) DCIS, (**D** and **E)** grade-2 and -3 IDCs, (**F**) grade-2 ILC, and (**G**) HER2, (**H**) basal/TNBC, and (**I**) luminal A subtypes of BC (*n* = 730, Supplemental Table 1). Panel **G** is shown also in Supplemental Figure 2, together with the negative staining control for the mAB DB144-N2 used in IHC (Supplemental Figure 2, M and N). Black arrowheads, epithelial BM; white arrowheads, ColXVIII absent in the epithelial BM; yellow arrowheads, single files of tumor cells in classic ILC; arrows, vascular BM; asterisks, cytoplasmic staining in tumor cells; a, adipocyte. Scale bars: 100 μm. (**J**–**O**) Kaplan-Meier plots showing RFS of patients with BC stratified by *COL18A1* mRNA expression levels (probe: 209082_s_at) by cancer grade (**J**–**L**) and by cancer subtype (**M**–**O**, also shown in Supplemental Figure 5 together with other survival data of BC subtypes). High ColXVIII expression, red line; low ColXVIII expression, black line. The open access gene expression data and patients’ survival information from The Cancer Genome Atlas (TCGA), the Gene Expression Omnibus (GEO) database, and the European Genome Archive (EGA), compiled in a single database at www.kmplot.com (22), were used for the meta-analyses. HRs and log-rank *P* values were computed using the median ColXVIII expression level as the cutoff. The initial number of patients in each group is indicated in the survival graphs.

Immunohistochemical (IHC) analysis of human BC specimens (Supplemental Table 1) with a custom-made monoclonal ColXVIII Ab (DB144-N2) (Supplemental Table 2 and Supplemental Figure 1B) showed that the ColXVIII signal was prominent in the BMs of blood vessels, mammary ducts, and lobules in normal breast tissue adjacent to the tumor regions (Figure 1A and Supplemental Figure 2). In addition, ColXVIII is detected in the thin BM surrounding the adipocytes. In ductal carcinoma in situ (DCIS), the ducts filled with tumor cells are usually surrounded by an intact ColXVIII^+^ BM/myoepithelial cell layer, albeit the ColXVIII signal may be discontinuous or even completely lacking at some tumor borders (Figure 1, B and C, and Supplemental Figure 2). Cytoplasmic ColXVIII staining ranging from weak to moderate could frequently be detected in tumor cells in DCIS (Figure 1, B and C, and Supplemental Figure 2), whereas the epithelial cells in normal and hyperplastic mammary glands (MGs) did not show cytoplasmic ColXVIII expression (Figure 1A and Supplemental Figure 2, A, B, G, and H).

In invasive ductal carcinomas (IDCs) of various grades, ColXVIII expression was commonly seen in the cytoplasm of tumor cells, although the staining intensity varied from weak to strong between samples and tumor regions and was more intense in high-grade tumors (Supplemental Table 3A). In IDCs, ColXVIII expression was either fragmented or completely lost from the epithelial BM/myoepithelium (Figure 1, D and E). In invasive lobular carcinoma (ILC), the most common special histological type of BC (21), ColXVIII often localized in tumor cells organized in linear, single-file chains of cells, and the signal intensity was mostly weak or moderate (Figure 1F, Supplemental Figure 2F, and Supplemental Table 3B). The ColXVIII signal was prominent in the vascular BMs of all DCIS, IDC, and ILC samples (Figure 1, A–I and Supplemental Figure 2, C–M). Occasionally ColXIII could also be detected in other stromal cells, including myofibroblasts (Supplemental Figure 2E). We established the authenticity of the ColXVIII staining patterns in several tumor samples with a custom-made polyclonal human ColXVIII Ab (QH48.18) (Supplemental Table 2, Supplemental Figure 1B, and Supplemental Figure 2, I–L).

We observed interesting patterns in ColXVIII expression when BC samples were classified according to their molecular subtypes. The cytoplasmic ColXVIII signal was usually strong or moderate in HER2 and basal/TNBC cases, and the staining intensity in the BM/myoepithelial cell layer varied from negative to strong (Figure 1, G and H, and Supplemental Table 3A). In some samples, ColXVIII was markedly upregulated in the cytoplasm of invading tumor cells, whereas the cytoplasmic ColXVIII signal at the DCIS site was weak, although both the surrounding BM/myoepithelium and the endothelium showed strong ColXVIII staining (Figure 1G). In luminal A samples, ColXVIII signals were variable in both the cytoplasm and around the tumor nests, ranging from negligible to prominent staining (Figure 1I and Supplemental Table 3A).

Analyses of open databases (22) for a potential association of *COL18A1* mRNA levels with survival of patients with BC showed that high *COL18A1* expression is significantly associated with poor prognosis in patients with high-grade BCs but not in unclassified patients or in those with low-grade tumors (Figure 1, J–O, and Supplemental Figure 4, A–F). When the patients with grade-3 cancers were further categorized into major molecular subtypes, ColXVIII significantly associated with poor relapse-free survival (RFS) in all subgroups (Figure 1, M–O, and Supplemental Figure 5). This was particularly evident in grade-3 HER2 cases, in which RFS rates were low despite the relatively small number of patients included in the analysis (Figure 1N and Supplemental Figure 5H). Survival analysis of patients with typical ILC (luminal A, estrogen receptor^+^, progesterone receptor^+^, HER2^–^) (21) did not show a significant association with ColXVIII expression (Supplemental Figure 4, G–I). Thus, the IHC and survival analyses linked high ColXVIII levels with high-grade tumors of “no special type” and, together with the IHC results, formed a solid foundation for additional studies especially in the HER2 and basal/TNBC subgroups of IDC.

We performed an in-house indirect ELISA assay to quantify the plasma levels of N-terminal ColXVIII fragments in a small number of healthy controls and patients with BC. The average plasma ColXVIII concentration in HER2 and TNBC subtypes, but not in luminal A types, was significantly higher than that in the controls (Supplemental Figure 6A). When the same data were grouped according to metastatic status, we found that plasma ColXVIII levels were significantly higher in lymph node^+^ than in node^–^ luminal A patients, suggesting that ColXVIII could predict metastasis in this subtype, whereas in the HER2 and basal/TNBC patients, ColXVIII could serve as an early diagnostic marker even before metastasis (Supplemental Figure 6, A and B).

### ColXVIII is coexpressed with EGFR and HER2 in human BC cells.

**Figure 2:**
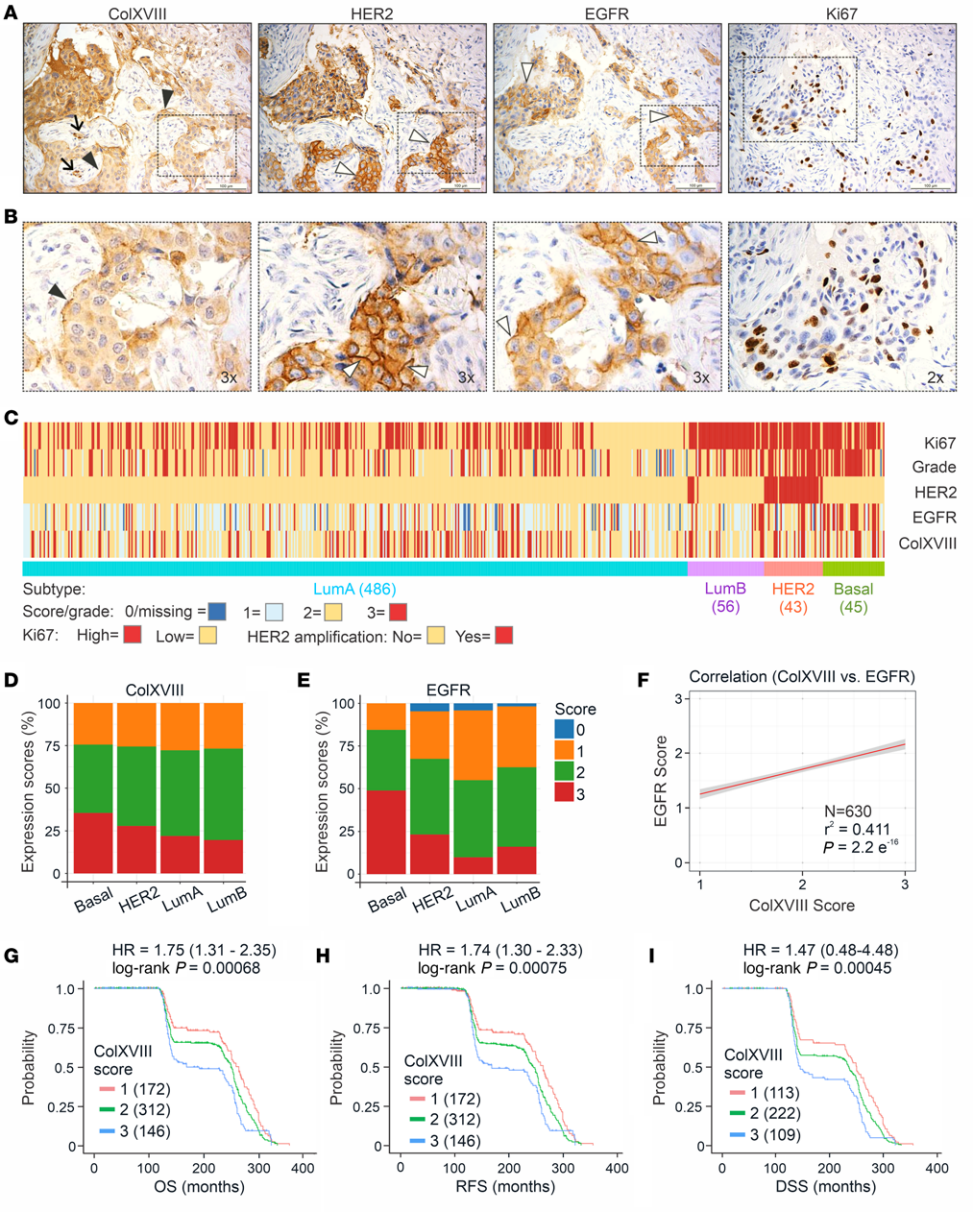
Expression of ColXVIII and routine BC biomarkers in human BC. (**A**) Representative images of IHC staining for ColXVIII, EGFR, HER2, and Ki67 in sequential sections of HER2 subtype BC (*n* = 70, Supplemental Table 1). Scale bars: 200 μm. Arrow, vascular BM; black arrowheads, epithelial BM; white arrowheads, EGFR or HER2 signal on the plasma membrane. (**B**) Magnified regions indicated in **A** (original magnification, ×2 and ×3). (**C**) Heatmap showing IHC scores for ColXVIII and EGFR, HER2 and Ki67 status, and nuclear tumor grades for BC samples from the Uppsala/Umeå cohort, grouped by BC subtypes. Samples analyzed for each molecular subtype: HER2, *n* = 43; luminal B, *n* = 56; luminal A, *n* = 486; and TNBC, *n* = 45. IHC scores and nuclear grades are indicated in different colors. (**D** and **E**) Proportions of IHC scores for ColXVIII and EGFR in BC subtypes in the Uppsala/Umeå cohort. (**F**) Correlation between the ColXVIII and EGFR scores in the Uppsala/Umeå cohort, calculated using the Kendall rank correlation coefficient (Kendall Tau). In **C**–**F**, *n* = 630. (**G**) Kaplan-Meier plots showing OS (*n* = 630), RFS (*n* = 630), and disease-specific survival (DSS) (*n* = 444) for the Uppsala/Umeå cohort of patients with BC, stratified by cytoplasmic ColXVIII protein expression levels. HR and log-rank *P* values were computed using the ColXVIII IHC scores as thresholds for stratification. The initial number of patients in each group is indicated in the survival graphs.

High *COL18A1* mRNA expression was associated with poor overall survival (OS) and RFS in HER2-amplified BC, with a HR above 1.8, (Figure 1N and Supplemental Figure 5, G–I). This notion prompted further investigations into the role of ColXVIII in ErbB signaling. Initial IHC analysis of HER2-type human BC specimens in the Oulu cohort (*n* = 21, Supplemental Table 1) showed that ColXVIII was highly expressed in HER2^+^ and EGFR^+^ tumor areas that had a high number of Ki67^+^ proliferating cells (Figure 2, A and B). We then analyzed the expression of ColXVIII and EGFR in a larger prospective Uppsala/Umeå BC tissue microarray (TMA) (*n* = 709) that had previously been scored for HER2 and Ki67 and the nuclear grade (Supplemental Table 1 and Supplemental Table 3C). After removing the samples with technically unacceptable ColXVIII staining from the analysis, approximately 73% of all BC cases (*n* = 630) showed strong (score 3) or moderate (score 2) cytoplasmic ColXVIII expression (Supplemental Table 3A and Supplemental Figure 3). When the grade-3 BC samples were classified into molecular subtypes, the proportion of cases with a high ColXVIII score were the biggest in the HER2 (~89%) and in the basal/TNBC (~86%) groups (Figure 2C and Supplemental Table 3A), indicating that prominent ColXVIII expression was associated with the aggressiveness of the BC. Furthermore, over 70% of all the BC samples with strong/moderate ColXVIII expression showed strong or moderate membranous EGFR expression (Figure 2, D and E, and Supplemental Tables 3, D and E), and a significant linear positive correlation between ColXVIII and EGFR scores was observed (Figure 2F). Finally, high ColXVIII protein expression by tumor cells was associated with poor prognosis and relapse in 78% of patients in the Uppsala/Umeå cohort (Figure 2, G–I, and Supplemental Table 3F), with a significant association found in the IDC group but not in the ILC group (Supplemental Figure 7). In summary, our results of both *COL18A1* mRNA expression in publicly available BC data sets and ColXVIII protein expression in BC tissue and plasma samples in independent patient cohorts demonstrate the association of high ColXVIII levels with the severity of disease and reduced survival, and with HER2 and EGFR expression.

### ColXVIII promotes tumor cell proliferation through its N-terminal TSP-1 domain.

**Figure 3:**
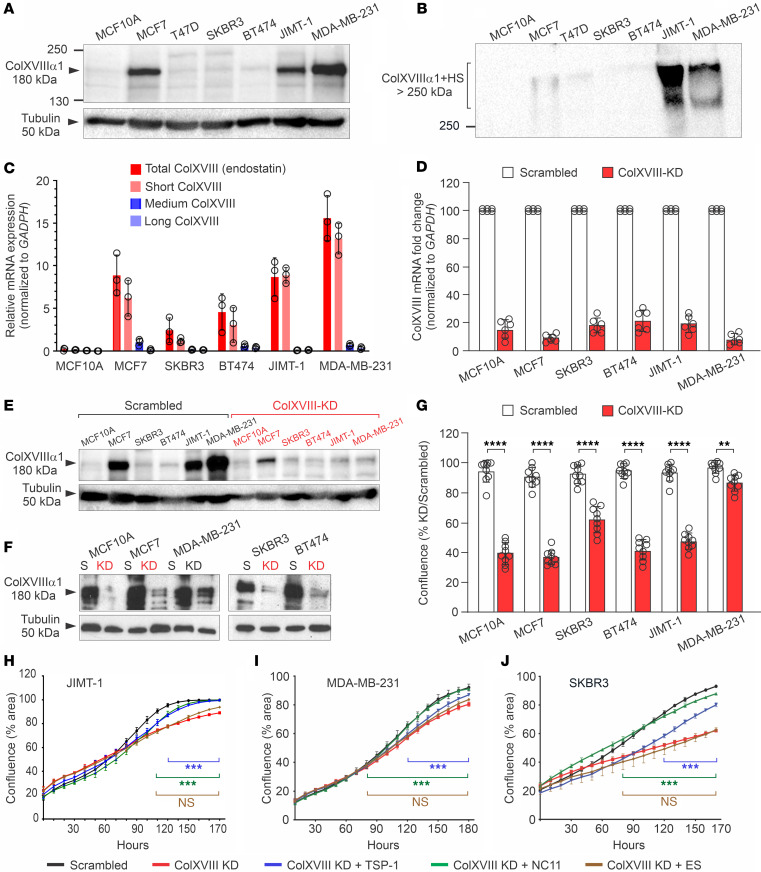
ColXVIII promotes BC cell proliferation through its N-terminal domain. (**A** and **B**) Representative immunoblots of ColXVIII in human BC and mammary epithelial cell lines. (**A**) In cell lysates, the size of the major ColXVIII band of approximately 180 kDa corresponds to the core polypeptide of the short isoform (60). (**B**) JIMT-1 and MDA-MB-231 cells secreted high amounts of glycosylated ColXVIII, which appears as a broad smear over 250 kDa. In **A** and **B**, biological replicates: lysates, *n* ≥5; culture media, *n* = 3. (**C**) Relative expression of the short, medium, and long *COL18A1* mRNA transcripts normalized to *GAPDH* in human BC cell lines (*n* = 3). The primer pairs are listed in Supplemental Table 5. (**D**) qRT-PCR analysis of total *COL18A1* mRNA after its KD in BC cell lines (*n* = 6). (**E** and **F**) Examples of immunoblots of ColXVIII protein levels in various KD cell lines and corresponding scrambled controls (S). *n* ≥3 biological replicates for each cell line. In **A**, **E**, and **F**, the loading control was β-tubulin. (**G**) Confluence of ColXVIII-KD versus scrambled cell lines (percentage), measured by an IncuCyte live-cell analysis system for 96 hours (*n* = 9). (**H**–**J**) Confluence of KD cell lines after administration of recombinant NC ColXVIII fragments (500 ng/mL) to the KD cells (*n* = 3). Untreated scrambled cell lines are shown as controls. TSP-1, TSP-1 domain; NC11, full-length N-terminal NC. Data in **C**, **D**, **G**, and **H**–**J** are presented as the mean ± SD. **P < 0.01, ***P < 0.001, and ****P < 0.0001, by 2-tailed Student’s *t* test (**C**, **D**, and **G**) and 2-way ANOVA with Dunnett’s multiple-comparison (treated vs. ColXVIII-KD) (**H**–**J**).

To validate in vitro BC models for further studies, we analyzed ColXVIII protein levels in several human BC cell lines and in 1 normal mammary epithelial cell line (Supplemental Table 4). Western blotting revealed prominent ColXVIII protein bands of approximately 180 kDa in HER2-amplified JIMT-1 and triple-negative MDA-MB-231 cell lysates, as well as in the MCF7 cells representing the luminal A subtype (Figure 3, A and E). In the other tested BC cell lines, namely T47D (luminal A), BT474 (luminal B), and SKBR3 (HER2), and in the noncancerous breast epithelial cell line MCF10A, we found that ColXVIII was also present but showed interexperimental variation (Figure 3, A, E, and F). Glycosylated ColXVIII, appearing as a smear with a molecular mass of more than 250 kDa, was abundant in the cell culture media of JIMT-1 and MDA-MB-231 cells, but less evident in the media of other BC cells (Figure 3B and Supplemental Figure 8A). A quantitative reverse transcription PCR (qRT-PCR) analysis showed that, in comparison with the nonmalignant MCF10A cell line, the short ColXVIII isoform was particularly overexpressed in human BC cells, up to 10-fold in JIMT-1 and 15-fold in MDA-MB-231 cells (Figure 3C).

To investigate the role of ColXVIII in breast carcinogenesis, we inhibited its expression in BC cells with RNA interference. A mixture of 2 siRNAs, targeting the N-terminal TSP-1 region and the C-terminal ES of ColXVIII (Supplemental Figure 1A), resulted in an efficient knockdown (KD) of ColXVIII in BC cell lines. Typically, a 70%–90% inhibition in mRNA synthesis was achieved by this approach as compared with the scrambled vector-transfected control cells, leading to a reduced amount of ColXVIII protein in cell lysates and in the culture media (Figure 3, D–F, and Supplemental Figure 8A). ColXVIII KD significantly reduced the proliferation of human BC cells, ranging from a reduction of approximately 10% in the MDA-MB-231 cells to a roughly 60% reduction in the other BC cells during a 96-hour follow-up period (Figure 3G). Thus, the KD experiments show that ColXVIII could support the proliferation of different types of BC cells, including those that express lower amounts of this collagen than JIMT-1 and MDA-MB-231 cells.

To confirm that the reduction in cell proliferation was caused by ColXVIII KD, and to determine which portion of the ColXVIII molecule could convey this effect, recombinant fragments of various NC domains of ColXVIII were added to BC cells, and proliferation was recorded up to 180 hours. JIMT-1 and MDA-MB-231 cells were chosen for this assay because they represent HER2 and basal/TNBC subtypes and were shown to secrete high amounts of ColXVIII. For comparison, we analyzed the HER2^+^ SKBR3 cells, which express less ColXVIII (Figure 3, A and B, and Supplemental Figure 8A). Both the TSP-1 fragment and the full-length N-terminal NC11 fragment (containing TSP-1, MUCL-18, and FZ-C18) (Supplemental Figure 1A) were able to reverse the inhibitory effect of siRNA-mediated ColXVIII depletion and restore the proliferation activity of the KD cells to the level of scrambled cells, especially in the HER2^+^ SKBR3 cell line (Figure 3, H–J). By contrast, ES could not rescue the reduced proliferation in any of the tested KD cell lines. Hence, the results of these in vitro experiments suggest that specifically the N-terminal portion of ColXVIII, and even the TSP-1 domain alone, can constitute an ECM signal that activates BC and mammary epithelial cell proliferation.

### ColXVIII supports mammary carcinogenesis in the MMTV-PyMT mouse model.

**Figure 4:**
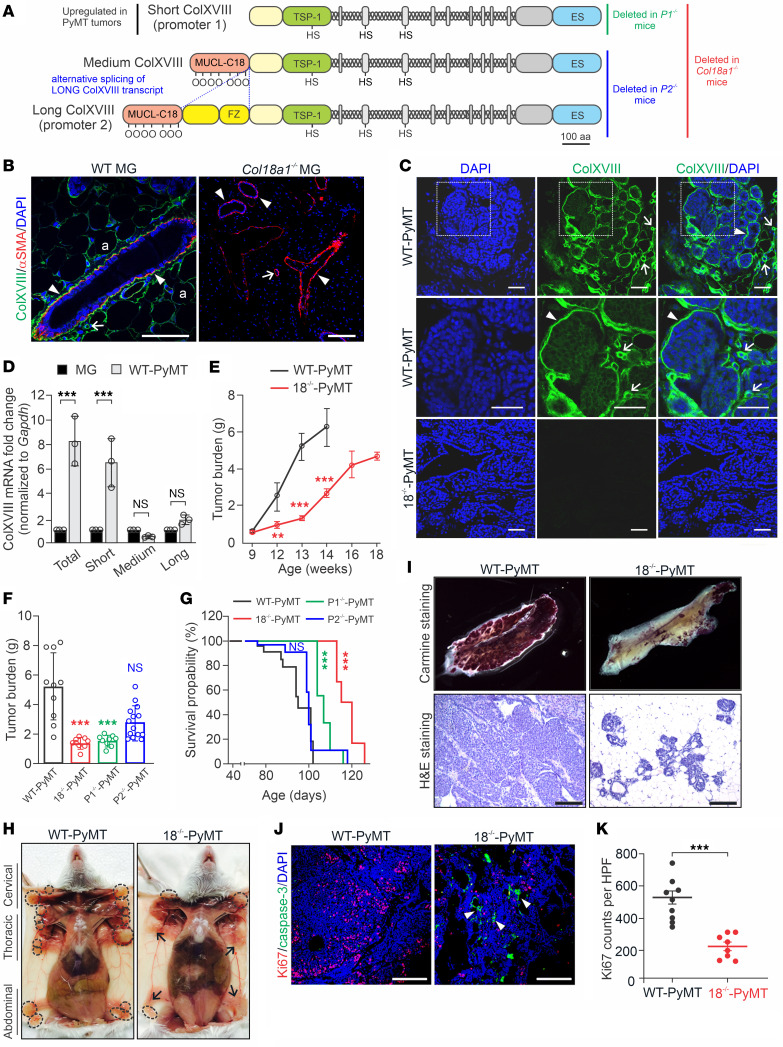
The short ColXVIII isoform promotes mammary tumor growth in mice. (**A**) Structures of 3 α1(XVIII) collagen chains and *Col18a1* mouse models. (**B** and **C**) ColXVIII expression (green) in MGs of WT and ColXVIII-KO (*Col18a1*^–/–^) females (*n* = 3) (**B**) and in WT MMTV-PyMT (WT-PyMT) and *Col18a1^–/–^* MMTV-PyMT (18^–/–^-PyMT) mammary tumors (*n* ≥10) (**C**). αSMA (red) in myoepithelial cells and vascular smooth muscle cells (**B**). Arrowheads, epithelial BM; arrows, vascular BM; a, adipocyte. (**D**) Expression of short, medium, and long *Col18a1* transcripts normalized to *Gapdh* in WT MGs and WT-PyMT tumors (*n* = 3). (**E**) Tumor burden in WT-PyMT and 18^–/–^-PyMT mice at 9–18 weeks of age (*n* ≥3 per genotype at each time point). (**F**) Mammary tumor burden at week 13 in WT-PyMT (*n* = 10), 18^–/–^-PyMT (*n* = 9), P1-PyMT (*n* = 9), and P2-PyMT (*n* = 14) mice. (**G**) Kaplan-Meier plots for WT-PyMT (*n* = 38), 18^–/–^-PyMT (*n* = 31), P1-PyMT (*n* = 28), and P2-PyMT (*n* = 33) mice. (**H**) Tumors (circles) in MGs of WT-PyMT and 18^–/–^-PyMT mice at week 13. Arrows indicate macroscopically normal MGs. (**I**) Carmine Alum-stained MGs and H&E-stained WT-PyMT and 18^–/–^-PyMT tumor sections at week 13 (*n* ≥6). (**J**) Ki67 (red) and cleaved caspase-3 (green) in WT-PyMT and 18^–/–^-PyMT tumors. Arrowheads point to apoptotic cells in the 18^–/–^-PyMT specimen. (**K**) Ki67^+^ cells in WT-PyMT (*n* = 9) and 18^–/–^-PyMT tumors (*n* = 8) (4 fields/tumor at ×20). Scale bars: 200 μm (**B**, **C**, **I**, and **J**). **P < 0.01 and ***P < 0.001, by 2-tailed Student’s *t* test (**D**, **E**, and **K**), 1-way ANOVA with Bartlett’s post-correction test for equal variances (**F**), and Mantel-Cox test (**G**). Error bars indicate the SEM.

We next investigated the role of ColXVIII in BC in vivo by crossing our *Col18a1* mouse models (Figure 4A) with the transgenic mouse mammary tumor virus–polyoma virus middle T antigen (MMTV-PyMT) mammary carcinogenesis model that recapitulates histological and molecular progression of human ductal BC, including upregulation of ErbB2 (23, 24). Consistent with the results of human tissue analyses, in healthy mouse mammary tissue, we observed that the ColXVIII signal resided around adipocytes, in vascular BMs, and in the mammary duct BMs, where it was located next to the α smooth muscle actin (αSMA), a marker of myoepithelial cells in mammary ducts and of smooth muscle cells in blood vessels (Figure 4B). As expected, the MGs and adipose tissue of healthy *Col18a1*^–/–^ mice were not reactive with the anti-ColXVIII Ab, whereas the αSMA signal was present in the ducts and vessels (Figure 4B). In WT MMTV-PyMT (WT-PyMT) mouse mammary tumor tissues, the ColXVIII signal was clearly increased and was abundant around tumor nests and in the vascular BMs (Figure 4C). In addition, cytoplasmic ColXVIII in tumor cells could be observed in PyMT lesions (Figure 4C).

A qRT-PCR analysis showed that the short ColXVIII isoform in particular was upregulated approximately 7- to 8-fold in WT-PyMT tumors in comparison with normal mouse mammary tissue (Figure 4D). To investigate more closely the expression and functions of distinct ColXVIII isoforms in mammary tumors, we established an 18^–/–^-PyMT mouse line lacking all the ColXVIII isoforms, a P1-PyMT line lacking only the short isoform, and a P2-PyMT line lacking the medium/long ColXVIII isoforms. Immunostaining of mammary tissues from these crosses confirmed that the ColXVIII signals in tumors, both around the tumor nests and in the tumor vasculature, resulted from the short isoform (Supplemental Figure 9A).

The overall tumor burden was markedly reduced in the 18^–/–^-PyMT mice by comparison with the WT-PyMT mice from week 10 onwards, the difference being statistically significant at weeks 12–14 (Figure 4, E and F). In line with the qRT-PCR analysis and immunostainings of PyMT tumor tissues that revealed the involvement of short ColXVIII in mammary carcinogenesis, the tumor burden was approximately 75% lower in both the P1-PyMT and 18^–/–^-PyMT females than in WT-PyMT females at week 13 (Figure 4, E and F). No further comparison between the groups was possible, since all the WT-PyMT mice reached the humane endpoint of the experiment by the age of 14 weeks, whereas the 18^–/–^-PyMT mice could be followed until week 18. The tumor burden in the P2-PyMT mice was slightly lower than that seen in the WT-PyMT group, but the difference was not statistically significant (Figure 4F). In agreement with these data, the survival rates of the 18^–/–^-PyMT and P1-PyMT mice were significantly better than those of the WT-PyMT and P2-PyMT mice (Figure 4G).

In the WT-PyMT mice, we observed tumors in all MGs, whereas in the 18^–/–^-PyMT mice, tumors were observed mainly in the cervical MGs (Figure 4H), probably due to faster development of tumors in the cervical and thoracic regions (24). Whole-mount carmine alum and the H&E stainings showed substantially less cancerous tissue in MGs of the 13-week-old 18^–/–^-PyMT mice than in those of the WT-PyMT mice, in which the MGs were filled with tumors (Figure 4I). Moreover, whereas most mammary tumors in the control mice transformed to carcinomas around week 10, and all of the WT-PyMT tumors could be classified as carcinomas at week 13, those in the 18^–/–^-PyMT mice transformed to carcinomas much later, around weeks 16–18, and presented as hyperplasia or adenomas at weeks 8–14 (Figure 4H and Supplemental Figure 9, B and C). We assessed tumor stroma by performing collagen I immunofluorescence and Masson trichrome stainings. There were no visible alterations in collagen I deposition between the WT-PyMT and 18^–/–^-PyMT tumors (Supplemental Figure 10A). In human BC cell lines, ColXVIII depletion did not cause significant changes in the expression of collagen I or other collagen types either (Supplemental Figure 8, B–D).

To investigate the cellular effects of *Col18a1* deletion on mammary carcinogenesis, tumor tissue samples collected from the WT-PyMT and 18^–/–^-PyMT mice were stained for the Ki67 proliferation marker and proapoptotic cleaved caspase-3. The average number of Ki67-^+^ cells was approximately 60% lower in the 18^–/–^-PyMT tumors than in the WT-PyMT tumors, indicating that cancer cell proliferation was compromised in the absence of ColXVIII (Figure 4, J and K). Clusters of caspase-3^+^ tumor cells were frequently detected in the 18^–/–^-PyMT tumors, whereas in the WT-PyMT tumors, their amount was negligible and could not be quantified (Figure 4J).

**Figure 5:**
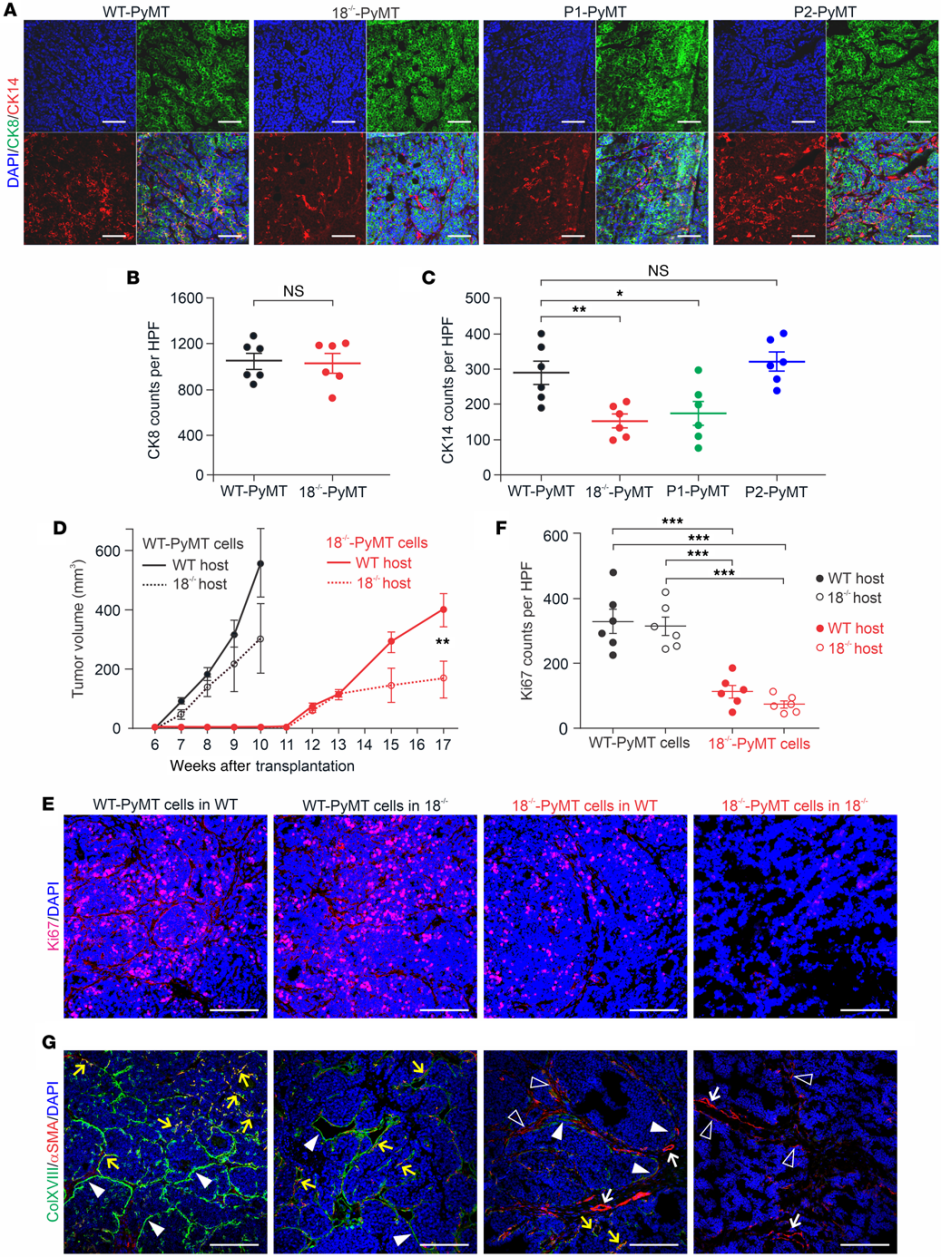
Expression of cytokeratins in PyMT tumors and orthotopic allograft transplantation experiments. (**A**) Representative images of immunostaining of CK8 and CK14 in WT-PyMT*,* 18^–/–^-PyMT, P1-PyMT, and P2-PyMT tumors. (**B**) Quantification of CK8^+^ luminal cells in WT-PyMT and 18*^–/–^*-PyMT tumors. (**C**) Quantification of CK14^+^ basal cells in WT-PyMT*,* 18^–/–^-PyMT*,* P1-PyMT, and P2-PyMT tumors. In **B** and **C**, *n* = 6/genotype; *n* = 4 random fields/tumor at ×20. (**D**) Growth rates of transplanted WT-PyMT and 18^–/–^-PyMT tumors in WT and *Col18a1*^–/–^ hosts. Number of mice (*N*) and allograft tumors (*n*): WT-PyMT cells in WT hosts and *18^–/–^-PyMT* cells in WT hosts (*N* = 12, *n* = 24), WT-PyMT cells in *Col18a1^–/–^* hosts and 18^–/–^-PyMT cells in *Col18a1*^–/–^ hosts (*N* = 6, *n* = 12). (**E**) Representative images of Ki67 immunostaining in WT-PyMT and 18^–/–^-PyMT allografts. (**F**) Quantification of the Ki67^+^ cell counts in transplanted tumors (*n* = 6/group, *n* = 4 random fields/tumor at ×20). (**G**) Representative images of ColXVIII (green) and αSMA (red) expression in allograft tumors. Arrowheads indicate ColXVIII^+^ structures or cells at tumor borders; open arrowheads show αSMA^+^ cells; yellow arrows point to αSMA and ColXVIII double-positive structures and cells; white arrows indicate αSMA^+^ blood vessels. Scale bars: 200 μm (**A**, **E**, and **G**). DAPI, blue. *P < 0.05, **P < 0.01, and ***P < 0.01, by 2-tailed Student’s *t* test (**B** and **D**) and 2-way ANOVA with Dunnett’s multiple-comparison test (**C** and **F**). Error bars indicate the SEM.

To study the identity of ColXVIII^+^ cells in MMTV-PyMT tumors, we performed immunostainings with the cytokeratin 14 (CK14) Ab specific for basal myoepithelial cells and with CK8 and CK18 Abs specific for luminal cells. ColXVIII expression was detected in both CK18^+^ luminal and CK14^+^ basal cells in mouse breast tumors (Supplemental Figure 10B). To further evaluate these cell types in our 4 PyMT models, we analyzed the numbers of CK8^+^ and CK14^+^ cells in tumor tissues (Figure 5A). We found equal numbers of CK8^+^ luminal cells in the WT-PyMT and the 18^–/–^-PyMT tumors but significantly, even 50%, less CK14^+^ basal cells in the 18^–/–^-PyMT tumors in comparison with the WT-PyMT tumors (Figure 5, B and C). In the P1-PyMT tumors, CK14^+^ basal cell counts were reduced as much as in the 18^–/–^-PyMT tumors, whereas in the P2-PyMT tumors, theCK14^+^ cells were as abundant as in the WT-PyMT tumors (Figure 5C). These observations suggest that the short ColXVIII had a role in sustaining the basal tumor cells in BC and tumor growth in the MMTV-PyMT model.

Finally, we observed a delay and impaired growth in pulmonary metastasis in the 18^–/–^-PyMT and P1-PyMT mice compared with the WT-PyMT and P2-PyMT mice. Hence, all the WT-PyMT and P2-PyMT mice sacrificed at the age of 13–15 weeks, and 90% and 60%, respectively, of those sacrificed at the age of 10–12 weeks had macrometastases in their lungs. By contrast, the 18^–/–^-PyMT and P1-PyMT mice developed lung metastases at later time points, so that only 10% of 18^–/–^-PyMT mice and 20% of the P1-PyMT mice had lung metastases at weeks 13–15, and 70% and 100%, respectively, at weeks 16–18 (Supplemental Figure 11, A and B). Overall, the WT-PyMT mice had the highest (95%) and the 18^–/–^-PyMT mice had the lowest (27%) proportion of lung metastases during the follow-up period (Supplemental Figure 11C). Image analysis revealed significantly larger tumor areas in the lungs of WT-PyMT and the P2-PyMT mice than in the lungs of 18^–/–^-PyMT and P1-PyMT mice (Supplemental Figure 11D).

### ColXVIII has an autocrine-stimulatory function in mammary carcinoma cells.

Reciprocal orthotopic allograft transplantations between the WT and *Col18a1*^–/–^ genotypes were performed to determine whether the tumorigenic functions of ColXVIII are tumor cell autonomous or microenvironmental. Both the WT and *Col18a1*^–/–^ female mice that received WT-PyMT tumor cells developed palpable tumors by week 7 after implantation (Figure 5D), but these grew faster in the WT hosts, reaching the humane endpoint size limit, on average 550 mm^3^, by week 10. In the *Col18a1^–/–^* hosts, the tumors were on average half the size of those in the WT hosts by the same week, and although the difference was not statistically significant, this observation suggested that host-derived ColXVIII could also have contributed to the regulation of tumor growth. Cells isolated from the 18^–/–^-PyMT tumors grew much more slowly, and palpable tumors developed 5 weeks later, at week 12, in both hosts. The 18^–/–^-PyMT tumors grew faster in the WT mice than in the *Col18a1*^–/–^ mice, reaching a size of approximately 400 mm^3^ in 17 weeks in the WT hosts and approximately 200 mm^3^ in the *Col18a1*^–/–^ hosts (Figure 5D). Ki67 immunostaining of the allografts showed that in both hosts, the implanted 18^–/–^-PyMT tumor cells proliferated significantly less than did the WT-PyMT cells (Figure 5, E and F). There was no statistical difference in the Ki67 scores for either the 18^–/–^-PyMT cells or the WT-PyMT cells between the hosts (Figure 5F).

Immunostaining of the WT-PyMT tumor allografts showed prominent ColXVIII signals at the borders of the tumor nests in the WT hosts but somewhat weaker signals at these sites in the *Col18a1*^–/–^ hosts (Figure 5G). At some sites, these signals overlapped with the αSMA present in the myoepithelial/endothelial cell layer of the tumor border. ColXVIII signals were rare when 18^–/–^-PyMT cells were injected into the WT host, although occasionally faint, discontinuous ColXVIII staining could be observed at tumor borders in the vicinity of αSMA^+^ stroma (Figure 5G). In 18^–/–^-PyMT tumors in the *Col18a1^–/–^* hosts, ColXVIII signals were completely lacking, and only αSMA^+^ myoepithelium and vasculature could be detected (Figure 5G).

### ColXVIII supports BC stem cells.

**Figure 6:**
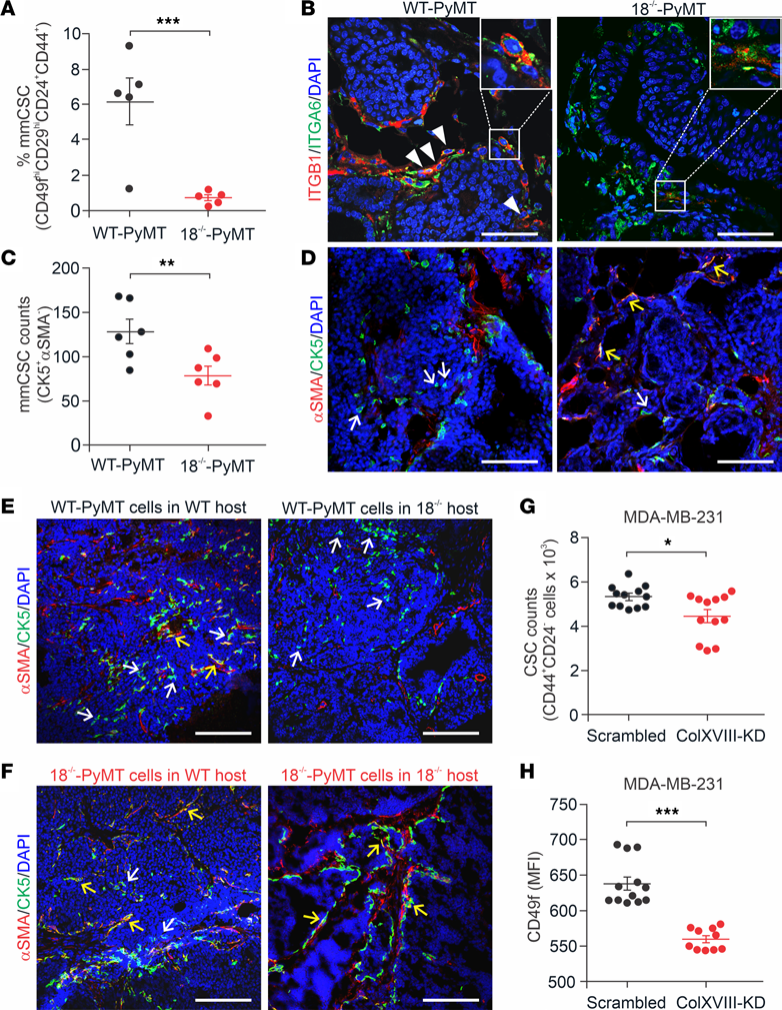
ColXVIII expression in mouse mammary CSCs. (**A**) Quantification of FACS-sorted CD44^+^, CD24^+^, CD29^hi^, and CD49f^hi^ mouse mammary CSCs (mmCSC) from tumors from 13-week-old WT-PyMT and 18^–/–^-PyMT mice (*n* = 5 per genotype; *n* = 3 technical replicates for each). (**B**) Representative images of ITGB1 (CD29, red) and ITGA6 (CD49f, green) immunofluorescence staining of mammary tumors at week 13. Arrowheads indicate CD29 and CD49f double-positive cells in WT-PyMT tumors. Insets show strongly double-positive cells in WT-PyMT tumors and weakly double-positive cells in 18^–/–^-PyMT tumors (*n* = 5). Original magnification of insets, ×2.5. (**C** and **D**) Analysis of CK5 and αSMA expression in WT-PyMT and 18^–/–^-PyMT tumor tissues at week 13. (**C**) Quantification of discrete CK5^+^ and αSMA^–^ cells (*n* = 6 per genotype; *n* = 4 random fields/tumor at ×20). (**D**) Representative images of CK5 (green) and αSMA (red) staining. (**E** and **F**) Representative images of CK5 (green) and αSMA (red) staining in allograft tumors. White arrows show CK5^+^ and αSMA^–^ progenitor cells; yellow arrows point to CK5/αSMA double-positive mature myoepithelial cells (*n* ≥6). (**G**) CSC populations in the ColXVIII siRNA–transfected KD and scrambled vector–transfected control MDA-MB-231 cells, as estimated by FACS-sorted CD44^+^ CD24^lo/–^ cells. (**H**) Quantification of the MFI of CD49f^+^ cells in ColXVIII-KD and control MDA-MB-231 cells. *n* = 4 biological replicates; *n* = 3 technical replicates (**G** and **H**). Scale bars: 100 μm (**B**, **D**, **E** and **F**). DAPI, blue. *P < 0.05, **P < 0.01, and ***P < 0.001, by 2-tailed Student’s *t* test (**A**, **C**, **G** and **H**). Error bars indicate the SEM.

High ColXVIII expression has been observed in human mammary stem and progenitor cell populations (25) and in other tissue-specific stem cell niches (20). Using FACS, we found that the frequency of mouse mammary CSCs, defined as CD49f^hi^ (α6-integrin^hi^), CD29^hi^ (β1-integrin^hi^), and hyaluronan receptor CD44^+^, and heat-stable antigen CD24^+^ cell populations (26) was reduced by almost 90% in the 18^–/–^-PyMT tumors in comparison with the WT-PyMT tumors (Figure 6A). Immunostaining of tumor tissues showed a notable reduction in the β1-integrin signal in the 18^–/–^-PyMT tumors relative to the controls, and double-positive cells for β1 and α6 integrins were rare in the knockout (KO) tumors (Figure 6B). Our previous analysis revealed fewer CK14^+^ basal cells in 18^–/–^-PyMT and P1-PyMT tumors than in WT-PyMT and P2-PyMT tumors (Figure 5, C and D). CK5 is another marker of mammary basal cells and a marker of mature myoepithelial cells when it is coexpressed with αSMA, but discrete CK5^+^SMA^–^ cells are regarded as CSCs (27). Single-positive CK5^+^ cells were abundant inside the WT-PyMT tumor nests but were approximately 40% less frequent in the 18^–/–^-PyMT tumors (Figure 6, C and D), further confirming that there were fewer CSCs in KO tumors. Immunostaining of allograft tumors showed plenty of CK5^+^ and αSMA^–^ CSCs in WT-PyMT tumors grown in both WT and *Col18a1*^–/–^ hosts, whereas 18^–/–^-PyMT tumors had CK5^+^αSMA^+^ double-positive cells, indicating reduced CSC characteristics and myoepithelial differentiation of these cells (Figure 6, E and F).

We then performed FACS to analyze the CD44^+^ and CD24^lo/–^ CSC populations (28) in the basal WT and KD MDA-MB-231 human BC cells. We observed a significant reduction in the frequency of this CSC population and MFI levels of CD49f in the siRNA-based ColXVIII-KD cells when compared with the scrambled-treated MDA-MB-231 cells (Figure 6, G and H). Moreover, the common stem cell–related transcription factors *NANOG*, *SNAI1*, *SNAI2* (*SLUG*), and *SOX2* (29) were downregulated in the MDA-MB-231–KD cells (Supplemental Figure 8E).

### ColXVIII forms a complex with EGFR and α6-integrin and regulates ErbB signaling.

**Figure 7:**
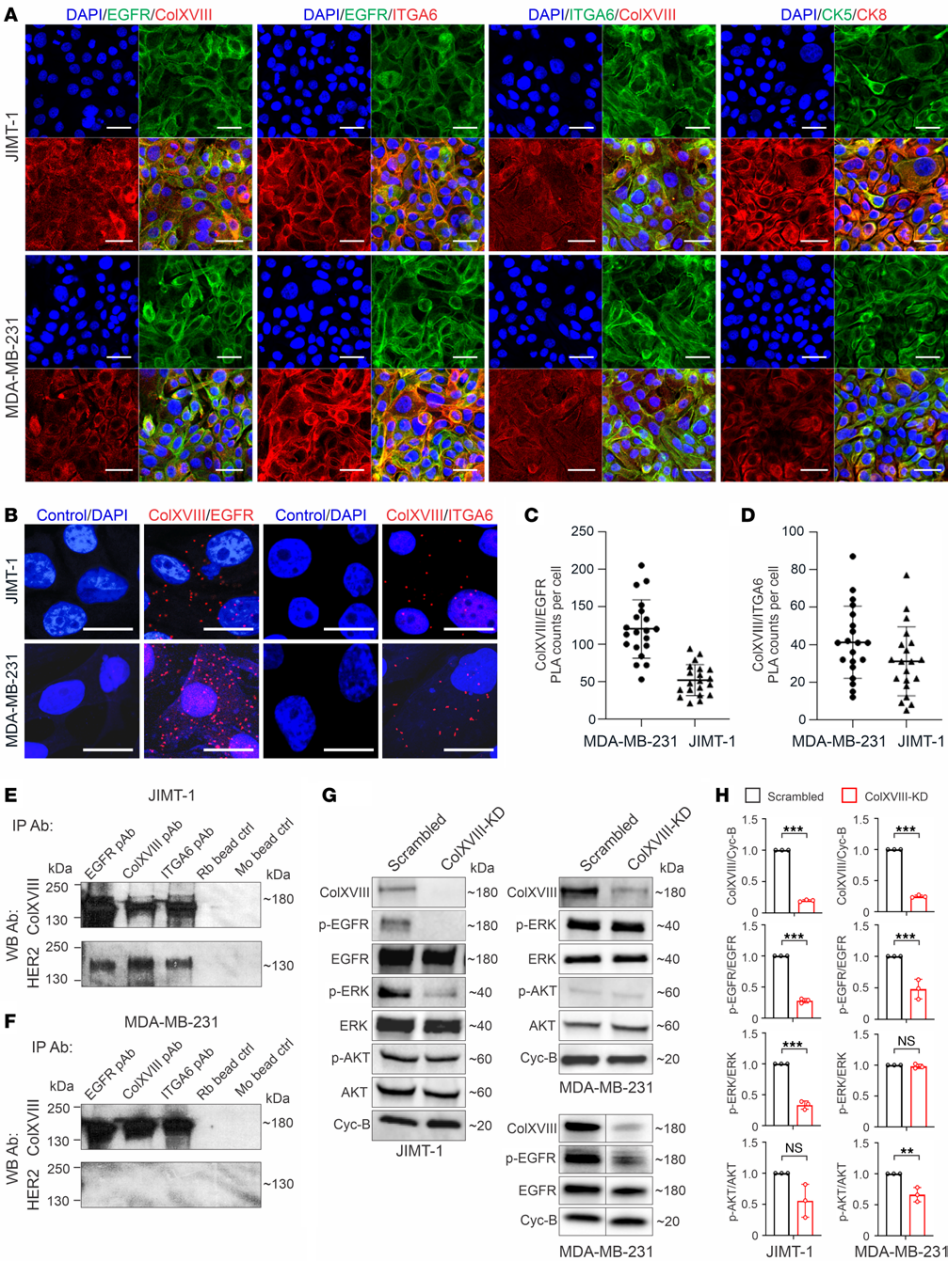
Interactions between ColXVIII, ErbBs, and integrins and analyses of ErbB signaling. (**A**) Representative images of immunostaining for ColXVIII, EGFR, and α6-integrin (ITGA6) in JIMT-1 and MDA-MB-231 cells (*n* ≥3). (**B**–**D**) In situ PLA in JIMT-1 and MDA-MB-231 cells. (**B**) Evidence of proximity (distance < 40 nm) for ColXVIII (mAb DB144-N2) and EGFR (mAb 52894) and for ColXVIII (mAb DB144-N2) and α6-integrin (primary Ab [pAb] 97760) is indicated by the presence of red dots. PLAs without pAbs served as the negative controls. Scale bars: 50 μm (**A**); 20 μm (**B**). (**C** and **D**) Quantitation of PLA counts for ColXVIII and EGFR (**C**) and ColXVIII and α6-integrin (**D**) (*n* = 3 biological replicates; *n* = 20 cells per sample). (**E** and **F**) Co-IP of ColXVIII (mouse mAB DB144-N2), EGFR (rabbit mAb 52894), and α6-integrin (rabbit pAb 97760) in HER2^+^ JIMT-1 (**E**) cells and in triple-negative MDA-MB-231 (**F**) cells. Protein complexes were detected in Western blot (WB) with ColXVIII (rabbit pAb QH48.18) and HER2 (rabbit mAb 4290) Abs (*n* ≥ 5). Goat anti–rabbit (Rb) IgG– and goat anti–mouse (Mo) IgG–coated magnetic bead controls are shown. (**G**) Representative immunoblots of EGFR and downstream signaling mediators in scrambled and ColXVIII-KD JIMT-1 and MDA-MB-231 cell lysates. In MDA-MB-231 cell lysates, the results for p-EGFR and EGFR along with ColXVIII were derived from another biological replicate. (**H**) Quantitation of ColXVIII, and EGFR, ERK, and AKT phosphorylation in JIMT-1 and MDA-MB-231 cell lysates (*n* = 3 biological replicates). **P < 0.01 and ***P < 0.001, by 2-tailed Student’s *t* test (**H**). Error bars indicate the SD.

Immunofluorescence showed ColXVIII and EGFR coexpression in HER2-amplified JIMT-1 and in basal-type MDA-MB-231 human BC cells (Figure 7A). Cooperation between EGFR and ECM receptor integrins promotes tumor progression and aggressiveness (14, 30). Therefore, we also examined the expression of α6- and β1-integrins, the key determinant of breast CSCs (31, 32), whose incidence was reduced upon ColXVIII ablation (Figure 6). IHC analyses revealed that α6-integrin was expressed with ColXVIII and EGFR in JIMT-1 and MDA-MB-231 cells (Figure 7A and Supplemental Figure 12). Proximity ligation assays (PLAs) demonstrated potential interactions between ColXVIII and EGFR and between ColXVIII and α6-integrin in both cell lines (Figure 7, B–D). Consistent with the PLA data, co-IP assays showed that EGFR and α6-integrin Abs pulled down ColXVIII and HER2 in JIMT-1 cells (Figure 7E) and ColXVIII in MDA-MB-231 cells (Figure 7F); the ColXVIII Ab pulled down HER2 in JIMT-1 cells and EGFR in MDA-MB-231 and SKBR3 cells; and the EGFR Ab pulled down HER2 in SKBR3 cells (Supplemental Figure 13, B and C). Neither EGFR nor ColXVIII Abs pulled down the β1-integrin subunit in MDA-MB-231 cells (Supplemental Figure 13D).

To investigate the mechanism of ColXVIII in ErbB signaling in BC, we assessed the expression of the ErbBs and downstream signaling mediators in ColXVIII-KD BC cell lines. Western blot analyses showed that EGFR phosphorylations were significantly decreased in JIMT-1, MDA-MB-231, and SKBR3 cells upon ColXVIII KD relative to the scrambled cells (Figure 7, G and H, and Supplemental Figure 13, E–G). Moreover, phosphorylated ERK (p-ERK) levels were significantly decreased in JIMT-1 and SKBR3 cells, as were p-AKT levels in SKBR3 cells (Figure 7, G and H, and Supplemental Figure 13, E and F), whereas in the JIMT-1 cells, which have an activating mutation in the *PI3KCA* subunit (33), it is likely that KD of ColXVIII did not affect the level of p-AKT, but the p-ERK signal was significantly lower than in the controls (Figure 7, G and H). Quantification of Western blot signals revealed that the ratio of p-AKT/AKT was significantly lower in MDA-MB-231–KD cells than in control cells, whereas the p-ERK/ERK ratio was not affected (Figure 7H), probably because of activating mutations in *KRAS* and *BRAF* in this cell line (34).

### Depletion of ColXVIII improves the efficacy of ErbB-targeting therapeutics.

**Figure 8:**
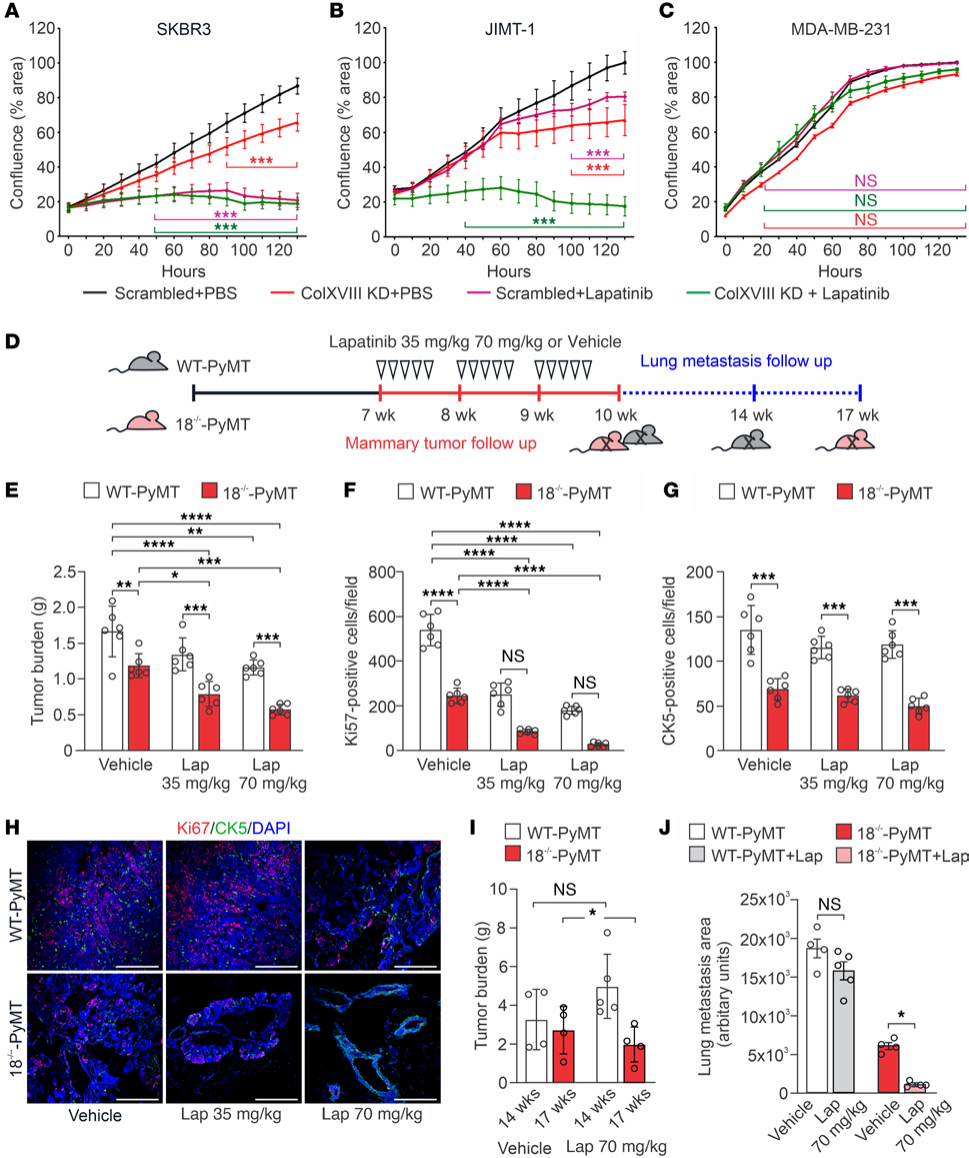
Depletion of ColXVIII improves the efficacy of ErbB-targeting drugs in preclinical BC models. (**A**–**C**) Cell proliferation, monitored as the cell confluence (percentage) for 5 days using the IncuCyte live-cell imaging platform, in BC cell lines with ColXVIII KD and lapatinib treatment (*n* = 3 biological replicates per cell line in triplicate). (**D**) Schematic figure of the lapatinib treatment regimen and follow up of the primary tumor growth (red) and lung metastasis (blue) in WT-PyMT and 18^–/–^-PyMT mice. (**E**) The total tumor burden in vehicle-treated (0.5% hydroxymethyl-cellulose) and lapatinib-treated WT-PyMT mice and 18^–/–^-PyMT mice at the age of 10 weeks. Two doses of lapatinib, 35 mg/kg and 70 mg/kg, were tested (*n* = 6 mice/group). (**F** and **G**) Quantification of the Ki67^+^ and the CK5^+^ cells (*n* = 6, *n* = 4 random fields/tumor at ×20). (**H**) Representative images of proliferating Ki67^+^ cells (red) and CK5^+^ mammary progenitor cells (green) in the vehicle- and lapatinib-treated WT-PyMT and 18^–/–^-PyMT tumors at week 10. Scale bars: 200 nm. (**I**) Mammary tumor burden in vehicle- and lapatinib-treated (70 mg/kg) WT-PyMT mice (14 weeks) and 18^–/–^-PyMT (17 weeks) mice (*n* = 4–5 mice/experimental group). (**J**) Quantification of lung metastasis area of vehicle and lapatinib (70 mg/kg) treated WT-PyMT mice (14 weeks) and 18*^–/–^*-PyMT (17 weeks) mice using ImageJ software (NIH) (*n* = 4–5). *P < 0.05, **P < 0.01, ***P < 0.001, and ****P < 0.0001, by 2-way ANOVA followed by Bonferroni’s post test (**A**–**C**) and Tukey’s multiple-comparison test (**E**–**G**, **I**, and **J**). Error bars indicate the SEM.

In view of these results, we finally focused our interest on the potential effects of ColXVIII inhibition on drug responses when combined with the SMI lapatinib or with humanized mAbs against HER2 (trastuzumab) and EGFR (panitumumab). Lapatinib treatment almost completely blocked the proliferation of the HER2-type SKBR3 cells, and thus ColXVIII KD, which by itself resulted in a roughly 30% reduction in cell proliferation within 5 days, did not yield any additional effect (Figure 8A). SKBR3 cells responded well to HER2-targeting trastuzumab, which alone led to an approximately 25% reduction in cell proliferation within 5 days. Interestingly, simultaneous administration of ColXVIII-targeting siRNAs and trastuzumab had a synergistic effect on SKBR3 cells, leading to a more than 60% reduction in cell numbers during the experiment as compared with vehicle-treated scrambled cells (Supplemental Figure 14A). EGFR-targeting panitumumab and ColXVIII siRNAs in combination inhibited the proliferation of SKBR3 cells more rapidly and efficiently than did either of these treatments alone (Supplemental Figure 14B).

HER2-amplified JIMT-1 cells are resistant to drugs that directly target ErbB receptors because of several coexisting drug resistance mechanisms, including mutations in *PI3KCA* that activate the PI3K/AKT pathway (33). We noticed that both lapatinib and ColXVIII KD alone led to growth inhibition only in the later stages of the follow-up period, but their combined effect was extremely rapid and efficient and almost completely abolished the proliferation of JIMT-1 cells (Figure 8B). Lapatinib, panitumumab, and trastuzumab treatments alone did not affect the proliferation of MDA-MB-231 cells because this cell line is HER2^–^ and has mutations in the downstream effectors *KRAS* and *BRAF* of the EGFR pathway that keep the cells in a proliferative state (34) (Figure 8C and Supplemental Figure 14, C and D). The EGFR-targeting panitumumab, however, did result in significant growth restriction in MDA-MB-231–KD cells, although the effect of ColXVIII inhibition was less impressive in this cell line than in the SKBR3 cells (Supplemental Figure 14, B and D). Besides these 3 cell lines, the HER2^+^ luminal B-type BT474 cell line that has a *PIK3CA* mutation (35) and is thus resistant to ErbB-targeting drugs was included in our tests. The proliferation of BT474 cells was not affected at all by trastuzumab, and only marginally by lapatinib. Depletion of ColXVIII KD alone reduced the proliferation of BT474 cells by 25%–30% within 5 days and sensitized these cells to lapatinib (Supplemental Figure 14, E and F).

Besides reducing cancer cell proliferation, ColXVIII KD significantly slowed down the migration of SKBR3 cells, but, as in the proliferation assay, it did not exert any additional inhibitory effect on wound closure when combined with lapatinib (Supplemental Figure 14G). In MDA-MB-231 cells, the inhibitory effect of ColXVIII KD was more evident in cell migration than in cell proliferation (Supplemental Figure 14H, and Figure 8C), whereas lapatinib produced only a marginal effect, as was expected, given the mutations in signal mediators (Supplemental Figure 14H). The combined use of lapatinib and ColXVIII siRNAs, but not single treatments with these reagents, resulted in a significant reduction of JIMT-1 cell migration (Supplemental Figure 14I).

A preclinical in vivo experiment with lapatinib (Figure 8D) confirmed that ColXVIII KO added a significant inhibitory effect on mammary carcinogenesis in the MMTV-PyMT mouse model. In the vehicle-treated 18^–/–^-PyMT mice, the total tumor burden was approximately 30% smaller than in the vehicle-treated WT-PyMT mice (Figure 8E). The tumor burden was further reduced in the 18^–/–^-PyMT mice treated with lapatinib, by approximately 35% and 54% compared with the vehicle-treated 18^–/–^-PyMT group, depending on the dose. Immunostaining showed that, while the mammary tumors of vehicle-treated WT-PyMT mice had high numbers of proliferative Ki67^+^ tumor cells, the numbers of dividing tumor cells were significantly lower in the vehicle-treated 18^–/–^-PyMT mice and in the lapatinib-treated WT-PyMT mice, and particularly low in the lapatinib-treated 18^–/–^-PyMT mice (Figure 8, F and H). Correspondingly, the tumors in the lapatinib-treated 18^–/–^-PyMT mice were considerably smaller (Figure 8E). Some of the 18^–/–^-PyMT mice receiving a high dose of lapatinib even had fairly normal-looking ductal structures in their fat pads (Figure 8H). The number of intratumoral CK5^+^ progenitor cells in 18^–/–^-PyMT tumors was initially significantly smaller than the number in WT-PyMT tumors (Figure 8, G and H, and Figure 6C), and lapatinib treatment did not affect the CK5 cell counts in either genotype in the current experiment (Figure 8G). Extended follow-up of the lapatinib-treated WT-PyMT mice resulted in a considerable increase in tumor burden following primary tumor relapse, and the humane endpoint was reached in 14 weeks. However, lapatinib treatment in the 18^–/–^-PyMT mice markedly reduced both primary tumor burden and lung metastasis compared with its control group (Figure 8, I and J, and Supplemental Figure 11E). In summary, our preclinical experiments demonstrate the importance of ColXVIII for BC cell functions and show that the inhibition of its action in tumor cells has promising therapeutic potential.

## Discussion

This study shows that ColXVIII expression is high in human and mouse BC and supports tumor cell proliferation in an autocrine manner through a mechanism involving ErbB signaling. The concept of integrated signaling through growth factor and ECM receptors is well established, and the downstream pathways of the 2 signaling systems overlap inside the cells (36). In line with this, our study demonstrates that ColXVIII was coexpressed and formed a complex with ErbB receptors and α6-integrin in BC cells, thus having the potential to facilitate MAPK/ERK and PI3K/AKT signaling and tumor cell proliferation and migration (Figure 7 and Figure 3, G–J). At present, however, experimental data are lacking that would demonstrate the direct binding between ColXVIII and EGFR/HER2 receptor pair and/or α6-integrin. One viewpoint is that ColXVIII could coordinate the growth factor and ECM receptor signaling events. This concept is further supported by the STRING (Search Tool for the Retrieval of Interacting Genes/Proteins) interaction analysis, which predicted functional associations between ColXVIII, ErbBs, and α6-integrins in epithelial cells (Supplemental Figure 13H).

The short isoform was found to be upregulated in BC (Figure 3C and Figure 4D) and involved in the revealed protumorigenic action of ColXVIII. The specific deletion of this isoform significantly inhibited cancer cell proliferation, primary tumor burden, and lung metastasis (Figure 4, E and F, and Supplemental Figures 9 and 11). Moreover, ectopic N-terminal TSP-1 partially halted the antitumorigenic effect caused by ColXVIII KD in human BC cells, whereas C-terminal ES did not promote tumorigenesis (Figure 3, H–J). Therefore, we believe that our finding of protumorigenic functions of ColXVIII and its TSP-1 domain will lead to further advancements in the field.

Most of the reported protein interactions for ColXVIII have been mapped to its C-terminal ES domain in endothelial cells and relate to angiogenesis regulation. These include binding of ES, for example, to α5- and αv-integrins and VEGF receptors (19, 20). We have recently shown that the N-terminal TSP-1 domain of ColXVIII interacts with α3β1-integrin in kidney tubule epithelial cells, where one of its functions is to regulate ureteric tree development (37). The present study suggested another perspective of ColXVIII interaction with α6-integrins in sustaining mammary CSCs (Figure 6, G and H). Further studies on the interaction network of ColXVIII would be of particular interest because the cooperation between α6β1 and α3β1-integrins was shown to regulate the progression and invasion of HER2 and basal/TNBC BCs (38, 39).

Collagens are currently being widely studied for their pivotal role in cancer progression, and it is interesting to compare our data on ColXVIII in BC with findings regarding the functionally diverse members of the collagen family. A recent bioinformatics analysis shows that fibrillar collagens I (*COL1A1*, *COL1A2*) and III (*COL3A1*), as well as *COL6A1*, *COL6A2*, *COL6A3*, *COL4A1*, *COL4A2*, *COL18A1* , and *COL12A1* are upregulated in BC (40). In agreement with this analysis, a proteomics-based analysis identified *COL1A1*, *COL1A2*, *COL3A1*, *COL6A3*, and *COL12A1* to be among the most abundant proteins in the ECM-enriched fraction of MDA-MB-231 xenografts (41). The study also showed that stromal cells are the main source of fibrillar collagens in this model and that BC xenografts of initially different metastatic potential differ in the expression of both tumor- and stroma-derived collagens. Interestingly, ColXVIII was abundantly secreted from the BC cells with high metastatic potential and, to an extent, compared with mouse stromal cells (41). It was suggested that BC cells of different metastatic capacity instruct local stromal cells to express preferred sets of collagens to remodel the TME for their needs. We propose that metastatic cells upregulate ColXVIII expression to support the formation of a favorable ECM for cell migration. This premise needs further investigation, but the current study already showed that in human BC, ColXVIII expression was particularly strong in collectively invading cells (Figure 1G) and in the invading CK14^+^ basal cells in MMTV-PyMT tumors (Supplemental Figure 10B), and that depletion of ColXVIII could also reduce the migration of both basal and luminal human BC cells (Supplemental Figure 14, G and H).

Multiple functional studies have clarified the role of collagen upregulation in BC. The seminal works using second harmonic generation microscopy revealed a network of aligned collagen fibers around advanced murine mammary tumors and demonstrated a causal relationship between stromal collagen I in tumor formation, collective tumor cell invasion, and metastasis (42, 43). By now, the contribution of large, linear, and highly cross-linked collagen fibers resulting in a stiff TME that activates mechanosignaling to promote carcinogenesis is established for BC (44–47). In contrast, the other major fibrillar collagen type III has been shown to restrict metastasis through modulation of the collagen I organization toward a complex or less-aligned form (48). Moreover, upregulation of *COL3A1* in disseminated tumor cells has been shown to shape the collagenous ECM architecture to induce and sustain dormancy of these cells (49). Several other collagen α chains, including *COL18A1*, were upregulated in dormant cells, but their inhibition did not disrupt tumor dormancy in vivo (49), further supporting the notion that high ColXVIII expression is implicated in tumor promotion.

Biochemical and biomechanical signals from the 3D ECM are implicated in the response to and resistance of cancer drugs (30, 50, 51). Mechanisms by which the inhibition of ColXVIII can overcome resistance to ErbB-targeting drugs (Figure 8 and Supplemental Figure 14) can be surmised through our own observations and data concerning other ECM molecules. For example, disruption of the interaction between laminin 332 and α6β4- or α3β1-integrins, and thereby the PI3K/AKT, MAPK/ERK1/2 and focal adhesion kinase (FAK) signaling and cell adhesion, sensitizes HER2^+^ BC cells to trastuzumab and lapatinib treatments (52). Moreover, high β1-integrin expression has been shown to predict a poor prognosis for trastuzumab- and lapatinib-treated HER2^+^ BC and induce resistance to these drugs through FAK and Src signaling (53, 54). Interestingly, ECM stiffness per se can reduce drug and radiation sensitivity in many cancers by forming a physical barrier against drug infiltration and by CSC promotion via various molecular mechanisms, including through integrin signaling (50, 55).

As a ubiquitous niche component, ColXVIII has roles in maintaining various types of tissue stem and progenitor cells (20). We have shown that in adipose tissue, the medium/long ColXVIII isoforms support the differentiation of progenitor cells and committed precursors to form mature adipocytes (56). Gupta et al. showed that ColXVIII is overexpressed in therapy-resistant breast CSCs, suggesting a role for it in the generation and propagation of these cells (25). Our work provided experimental evidence to support this finding. The number of cells with CSC characteristics was reduced in both mouse mammary tumors with *Col18a1* deletion and in human BC cells with reduced ColXVIII expression (Figure 6 and Supplemental Figure 8E). The demonstrated interaction between ColXVIII and α6-integrin in BC cells (Figure 7), a key integrin subunit in breast CSCs (26), is probably implicated in the maintenance of the stemness properties of BC cells. CSCs are not only responsible for sustaining primary tumors, but are also connected with the metastatic dissemination of neoplastic clones to distant organs (12–14), and we show here that both the primary tumor burden and lung colonization were reduced in mice with *Col18a1* depletion (Figure 4 and Supplemental Figure 11). Our previous work has shown that deletion of *Col18a1* leads to BM loosening and reduced stiffness (57). Thus, it is possible that ColXVIII upregulation in solid cancers may affect both the biomechanical and biochemical properties of the tumor ECM and the maintenance of CSCs and thereby regulates tumorigenesis and drug responses.

In conclusion, our findings indicate that signaling cues triggered by the N-terminal TSP-1 domain of short ColXVIII and transmitted through ErbBs and/or integrins can potentiate BC cell functions and promote the development of drug resistances, especially in advanced HER2-type BC (Figure 8B and Supplemental Figure 14F). The targeting of ColXVIII in the TME could therefore provide a therapeutic approach for achieving BC regression, even in cases in which the tumor does not show a response to the clinically tested drugs that inhibit ErbB signaling. Our data also show that ColXVIII could be of substantial value as a biomarker of BC progression, either scored in tissues or quantified in liquid biopsies.

## Methods

Details and sources of the research materials and experimental protocols are provided in the Supplemental Methods.

### Human BC samples and survival analysis.

Formalin-fixed, paraffin-embedded human BC tissue samples were from a patient cohort at Uppsala/Umeå University Hospital, Sweden (*n* = 709) (Supplemental Table 1) and assembled in a TMA in which each sample was represented as a duplicate. Expression of ColXVIII and EGFR was analyzed in the TMA, which had previously been scored for HER2 and Ki67 expression and the nuclear grade (Supplemental Table 3C) (58). Additional BC samples were from a cohort at Oulu University Hospital, Finland (*n* = 21), and these were stained for ColXVIII, EGFR, HER2, and Ki67. Circulating levels of ColXVIII were determined in 32 patients with BC from the Uppsala/Umeå cohort and in 6 healthy female volunteers using an indirect ELISA. The association of ColXVIII expression with BC patient survival was analyzed in open databases using the Kaplan-Meier survival analysis on the Kaplan-Meier Plotter (www.kmplot.com) (22) and R for the Uppsala/Umeå cohort.

### Mouse models.

A transgenic mouse mammary carcinogenesis model based on mammary tumor virus promoter–driven expression of the polyoma middle T antigen (MMTV-PyMT FVB/N) (24) was crossed with 3 *Col18a1*-KO models, all on the FVB/N background (56, 59). The origin and sources of animals are described in Supplemental Methods. Animal experiments were conducted at the Oulu Laboratory Animal Centre. Mammary tumor growth was monitored in randomized experimental groups of female mice at specific time intervals depending on the rate of tumor growth and the humane endpoint criterion as explained in Supplemental Methods. The number of animals in each experimental group ranged from a minimum of 3 mice at week 6 up to 14 mice at week 13. Lung metastasis was studied in experimental groups of mice aged 10–12 weeks, 13–15 weeks, and 16–18 weeks, with 10 mice per genotype in each group. An experiment of reciprocal orthotopic allograft transplantation between control and *Col18a1*-deficient mice (*n* = 6–12) was performed as described in Supplemental Methods. In vivo animal experiments were conducted in a nonblinded manner. Mouse tumor tissues were studied by histological and immunohistochemical, morphometric, flow cytometric, and qRT-PCR methods as described in Supplemental Methods. The number of samples and technical replicates for the quantitative analyses are indicated in the Figure legends.

### Cell studies.

Expression of ColXVIII in the human BC cell lines (Supplemental Table 4) was analyzed by qRT-PCR (see list of primers in Supplemental Table 5), Western blotting, and immunocytochemistry (see list of Abs in Supplemental Table 2). In vitro loss-of-function experiments were performed by siRNA-based KD of ColXVIII in BC cells, and gain-of-function experiments with ColXVIII-KD cells supplemented with recombinant ColXVIII fragments. Live-cell imaging was used for functional analyses of cell proliferation and migration. We examined the interactions of ColXVIII with ErbB and integrin receptors using proximity ligation and co-IP experiments, and cell signaling by assessing phosphorylation of EGFR and HER2 and the downstream mediators using Western blotting. Cell culture experiments were performed independently at least 3 times per experiment as indicated in the Figure legends.

### Drug tests.

To investigate the medical relevance of ColXVIII targeting, the ErbB inhibitors lapatinib, trastuzumab, and panitumumab were used in combination with ColXVIII KD in human BC cell lines cells and their respective controls. In vivo lapatinib treatment was applied to control and *Col18a1*-null PyMT mouse groups at 2 different doses to test the efficacy of the drug for primary tumor growth and lung metastasis.

### Statistics.

Statistical analyses were performed using the unpaired *t* test for experiments with 2 groups, and a 2-way ANOVA with Bonferroni’s post test when comparing data from experiments with multiple groups. A repeated-measures 1-way ANOVA was used to analyze the primary tumor growth curves (with Dunnett’s multiple-comparison test and Bartlett’s post correction test). The Kendall rank correlation coefficient (Kendall Tau) was used to analyze the association between ColXVIII and EGFR expression levels. Mouse survival analysis was performed using the log rank (Mantel-Cox) test. Differences were considered statistically significant at a *P* value of less than 0.05. GraphPad Prism (GraphPad Software) was used to conduct the statistical analyses.

### Study approval.

The use of human BC samples was approved by the ethics committee at the Medical Faculty of Umeå University (Dnr 09-175M); the regional ethics review board in Uppsala (Dnr 2005:118); and the ethics committee of the Northern Ostrobothnia Health Care District (Dnr 88/2000 and amendments Dnr 194/2013 and Dnr 100/2016). Informed consent for data use was obtained from all patients and volunteers prior to participation in the study. All human samples and clinical data were anonymized and labeled with a research code for blinded histopathological and plasma analyses. Only authorized personnel of the Oulu, Uppsala, and Umeå University Hospitals had access to personal and clinical data. Animal experiments were approved by the Finnish National Animal Experiment Board (permits ESAVI/6105/04.10.07/2015, ESAVI/1188/04.10.07/2016 and ESAVI-2936-04.10.07/2016).

### Data availability.

Values for all data points shown in graphs and values behind the reported means are provided in the Supplemental Supporting Data Values file. Participant data for the Uppsala/Umeå cohort are available upon request with a data transfer agreement. Participant data for the Uppsala/Umeå cohort are available upon request from MS (malin.sund@umu.se) under standard rules of protecting data integrity and existing ethical permissions.

## Author contributions

RD designed and performed all the experiments, analyzed the data, and wrote the manuscript. VI designed some experiments, analyzed the data, and provided scientific input. HP supervised the work, analyzed the human IHC data, and wrote the manuscript. GR, MRV, and TV scored the immunostainings of human samples. HR designed and performed some of the mouse experiments. SK provided the human BC samples and analyzed the human IHC data. GMN performed some of the mouse experiments. SMK optimized ColXVIII IHC for human samples. IK produced the recombinant proteins. JK provided scientific input. TS provided the mouse ColXVIII Ab. RW provided human tissue samples. AM provided scientific input. FW provided human samples and data and gave scientific input. MS provided human samples and data, gave scientific input, and wrote the manuscript. TP conceptualized and supervised the studies, provided scientific input, and wrote the manuscript. RH conceptualized and supervised the studies, provided scientific input, performed mouse tissue and cell analyses, and wrote the manuscript.

## Supplementary Material

Supplemental data

Supplemental table 3

Supporting data values

## Version 1 07/27/2023

In-Press Preview

## Version 2 09/15/2023

Electronic publication
