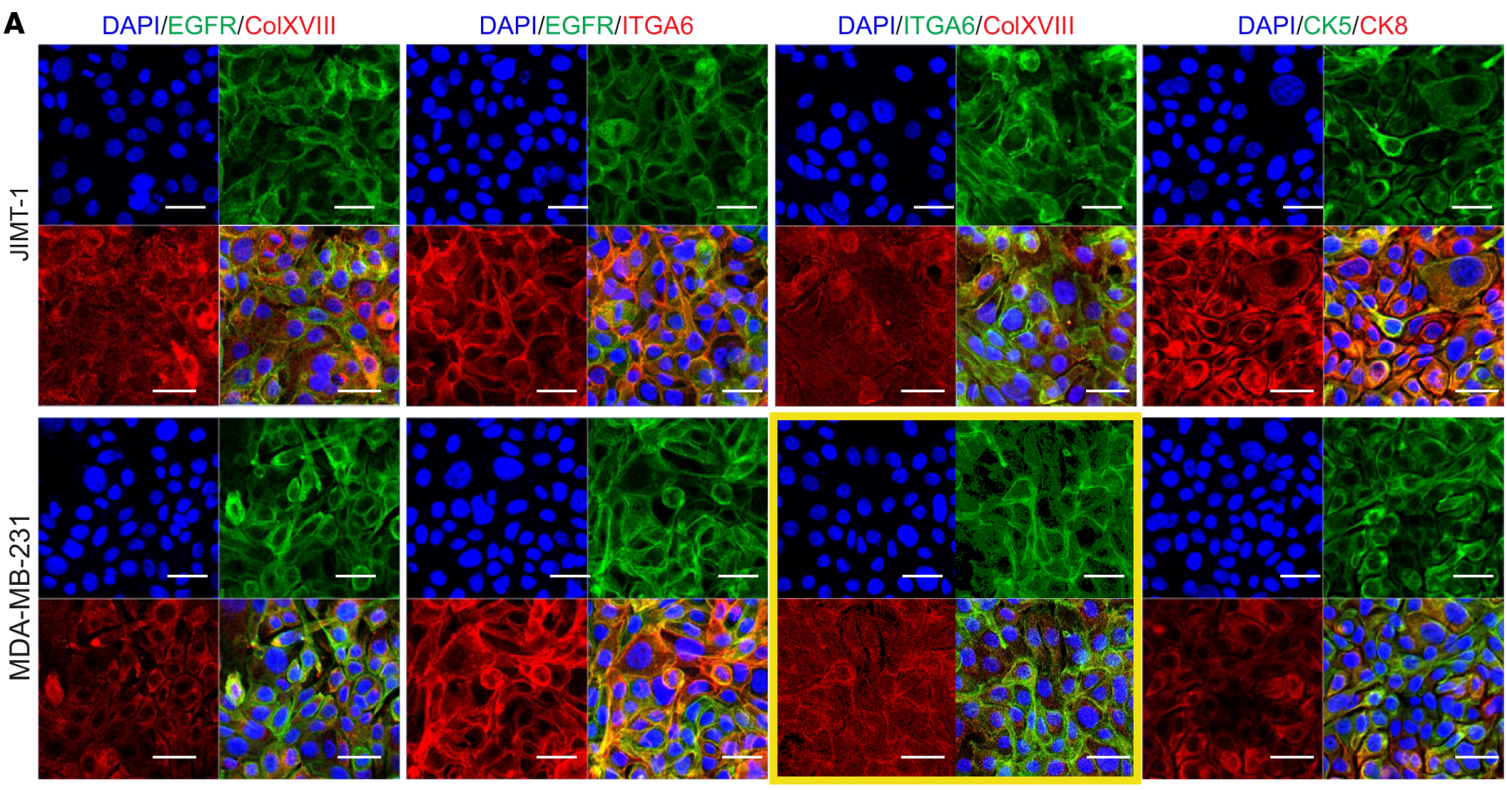# Corrigendum to Targeting collagen XVIII improves the efficiency of ErbB inhibitors in breast cancer models

**DOI:** 10.1172/JCI196478

**Published:** 2025-08-01

**Authors:** Raman Devarajan, Valerio Izzi, Hellevi Peltoketo, Gunilla Rask, Saila Kauppila, Marja-Riitta Väisänen, Heli Ruotsalainen, Guillermo Martínez-Nieto, Sanna-Maria Karppinen, Timo Väisänen, Inderjeet Kaur, Jussi Koivunen, Takako Sasaki, Robert Winqvist, Aki Manninen, Fredrik Wärnberg, Malin Sund, Taina Pihlajaniemi, Ritva Heljasvaara

Original citation: *J Clin Invest*. 2023;133(18):e159181. https://doi.org/10.1172/JCI159181

Citation for this corrigendum: *J Clin Invest*. 2025;135(15):e196478. https://doi.org/10.1172/JCI196478

In Figure 7A of the original article, there were errors in the DAPI/ITGA6/ColXVIII images shown for the MDA-MB-231 cell line. The authors determined that the incorrect images were serial images of JIMT-1 cells. The corrected figure, based on the original source data, is provided below. The HTML and PDF versions of the article have been updated.

The authors regret the errors.

## Figures and Tables

**Figure 7A:**