# Supplementary material for: Targeting collagen XVIII improves the efficiency of ErbB inhibitors in breast cancer models
Source: J Clin Invest. 2023 Sep 15;133(18):e159181. doi: 10.1172/JCI159181 (PMC10702473; doi:10.1172/JCI159181)
Supplement: Supplemental data [file jci-133-159181-s041.pdf]

SUPPLEMENTAL MATERIAL

Devarajan et al. Targeting collagen XVIII improves the efficiency of ErbB inhibitors in breast cancer models

Supplemental Figures

Figure 1. Schematic picture of the structure of ColXVIII and validation of human anti-ColXVIII antibodies
Figure 2. ColXVIII immunohistochemistry in human breast cancer and in normal mammary gland
Figure 3. Scoring of ColXVIII and EGFR protein expression in human breast cancer
Figure 4. *COL18A1* mRNA expression and breast cancer patient survival
Figure 5. *COL18A1* mRNA expression and patient survival in grade 3 breast cancers of different molecular subtypes
Figure 6. ColXVIII expression in breast cancer patients' plasma
Figure 7. Correlation between ColXVIII protein expression and survival of patients with invasive ductal breast cancer (IDC) or invasive lobular breast cancer (ILC)
Figure 8. ColXVIII knockdown (KD) in human breast cancer cell lines
Figure 9. Mammary carcinogenesis in MMTV-PyMT mice crossed with *Col18a1* mouse models
Figure 10. Expression fibrillar collagen, CK18 and CK14 and in mouse mammary tumors
Figure 11. Lung metastasis in MMTV-PyMT mice
Figure 12. Co-localization of ColXVIII, ErbBs and integrin α6 in mouse mammary tumors
Figure 13. Interactions between ColXVIII, ErbBs and integrins
Figure 14. Analysis of breast cancer cell proliferation and migration after ColXVIII knockdown and/or EGFR/ErbB-targeting drug treatment

Supplemental Tables

Table 1. Human breast cancer samples with histopathological and relapse data
Table 2. Antibodies
Table 3. ColXVIII and EGFR expression in the Uppsala/Umeå cohort (an Excel spreadsheet)
Table 4. Human breast cancer cell lines used in this study and their key characteristics
Table 5. qRT-PCR primers
Table 6. Statistical analyses (an Excel spreadsheet)

Supplemental Materials and Methods

Supplemental Figures

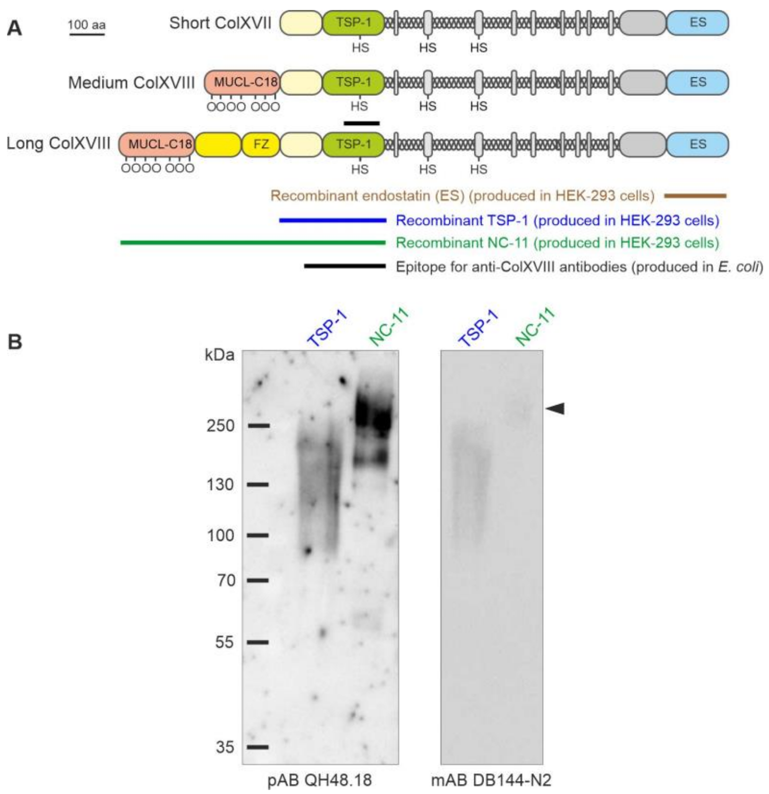

Supplemental Figure 1. Schematic picture of the structure of ColXVIII and validation of human anti-ColXVIII antibodies. (**A**) Domain organizations of three α1(XVIII) collagen chains (short, medium, and long). Abbreviations; NC, non-collagenous domain; TSP-1, thrombospondin-1; ES, endostatin; MUCL-18, mucin-like domain in ColXVIII; FZ, frizzled domain; O, O-glycan; HS, heparan sulphate; Scale bar, 100 amino acids. The picture also shows the N-terminal recombinant fragments used for Western blot in B, and the N- and C-terminal fragments used in the intervention experiments in Figure 3, H–J, as well as the epitope used for the generation of anti-ColXVIII antibodies (AB), the monoclonal (m) DB144-N2 (1) and the polyclonal (p) QH48.18 (2). (**B**) N-terminal recombinant fragments of mouse ColXVIII were expressed in HEK-293 cells, and purified from the conditioned culture media by metal ion affinity chromatography (ProBond Purification system, Invitrogen) and gel filtration in Superdex 200 column (GE Healthcare Life Sciences) as described previously (3, 4). Both the pAB QH48.18 (dilution 1:1000) and mAB DB144-N2 (dilution 1:500) recognized TSP-1 and NC-11 fragments but QH48.18 was more sensitive in the Western blot application. The predicted core polypeptide sizes of TSP-1 and NC-11 fragments are ~36 kDa and ~80 kDa, respectively. The HS chains in TSP-1, and HS chains plus O-glycans in NC-11 result in recombinant proteins with higher molecular weight, appearing in Western blot as broad smears of approximately 90-250 kDa and 190-300 kDa, respectively. Arrowhead, in the Western blot the NC-11 fragment gives much weaker signal with the mAB DB144-N2 than with the pAB QH48.18.

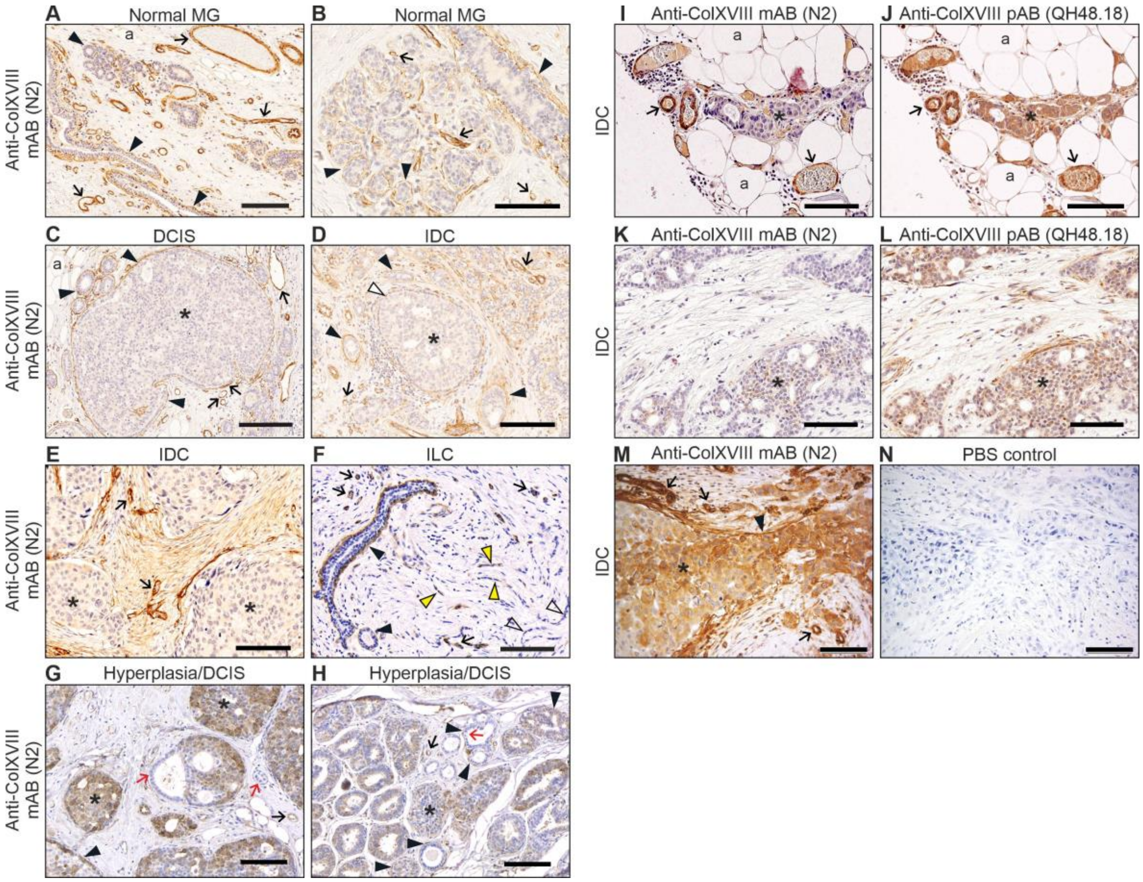

Supplemental Figure 2. ColXVIII immunohistochemistry in human breast cancer and in normal mammary gland. (**A–H**) A monoclonal anti-ColXVIII antibody (mAB) DB144-N2 (1) was used to detect ColXVIII in human breast tissues (total n=730). MG, normal mammary gland; DCIS, ductal carcinoma in situ; IDC, invasive ductal carcinoma; ILC, invasive lobular carcinoma. (**I–N**) Comparison of ColXVIII signals in sequential IDC sections stained with two anti-ColXVIII antibodies, the mAB DB144-N2 and the pAB QH48.18 antibody (2) (n=10 samples). (**M**) DB144-N2 staining of HER2 type IDC (also shown in the main Figure 1G) and (**N**) its negative control in which the primary antibody was replaced with PBS. Symbols: Black arrowhead, basement membrane (BM); white arrowhead, ColXVIII absent from the BM; arrow, vascular BM; asterisk, cytoplasmic ColXVIII staining in tumor cells; a, adipocyte; in **F**, yellow arrow, ColXVIII-positive single files in ILC; open arrow, ColXVIII-negative single files in ILC; in **G** and **H**, red arrow, ColXVIII-negative mammary epithelial cells in normal or hyperplastic ducts. Scale bars 100µm. Panel M is shown also in Figure 1G.

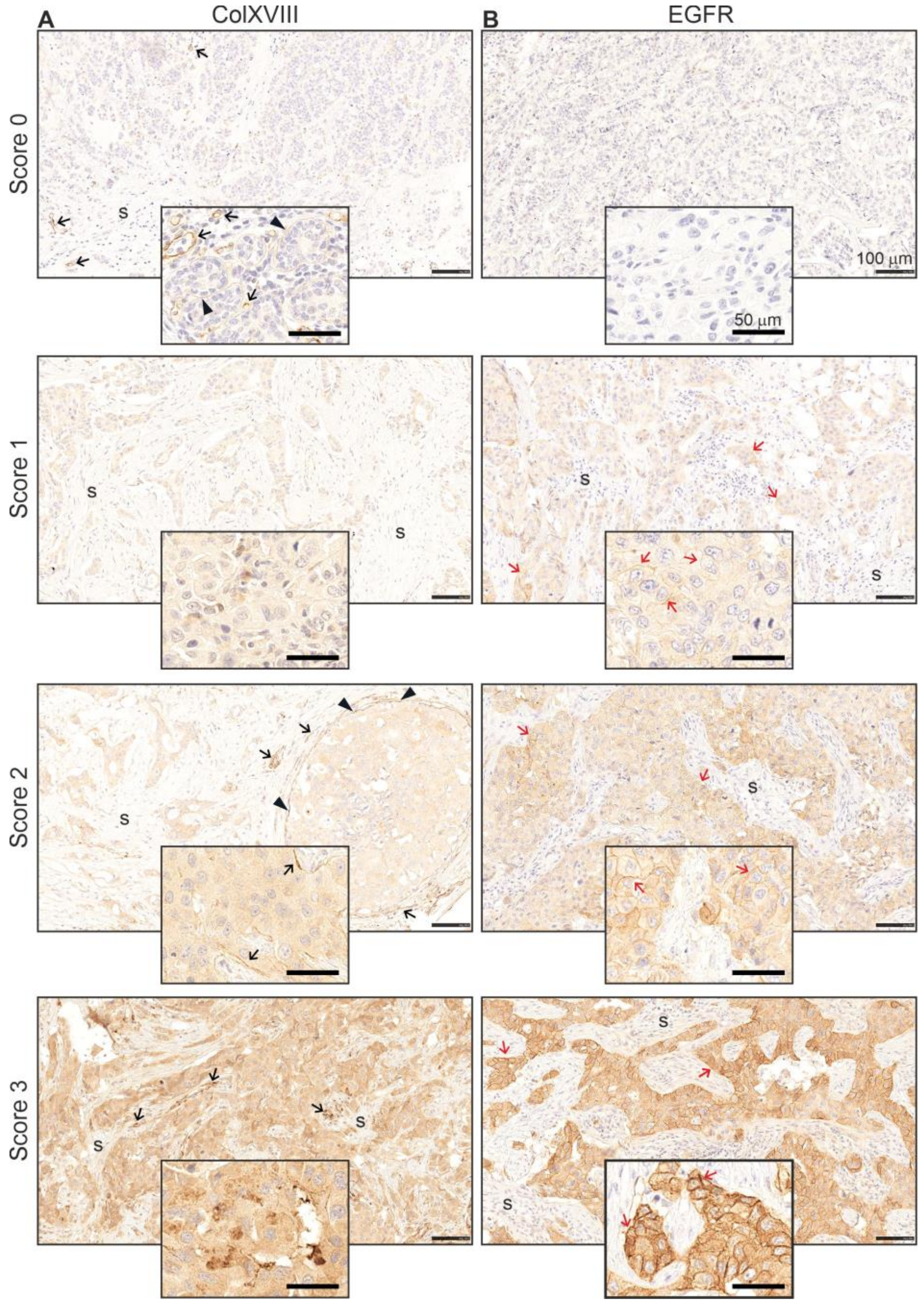

Supplemental Figure 3. Scoring of ColXVIII and EGFR protein expression in human breast cancer. Representative examples of human breast cancer (BC) specimens showing cytoplasmic ColXVIII (**A**) and membrane EGFR (**B**) expression scored as 0, negative; 1, weak; 2, moderate; and 3, strong. Examples of two different BC samples with two different magnifications are shown. Altogether 630 human BC samples were scored for ColXVIII and EGFR. Basement membrane (BM)/myoepithelial ColXVIII signal (arrowhead) was not considered in scoring because in tumors it is either negative, fragmented or continuous positive signal around different tumor nests, even in the same sample. EGFR shows positive signals both on the cell membrane and in the cytoplasm of tumor cells, but only the membrane EGFR was scored. In **A**, black arrow, capillary BM; s, tumor stroma; in **B**, red arrow, membrane EGFR. For all images, scale bars are 100µm and 50µm as marked in top in the panel B.

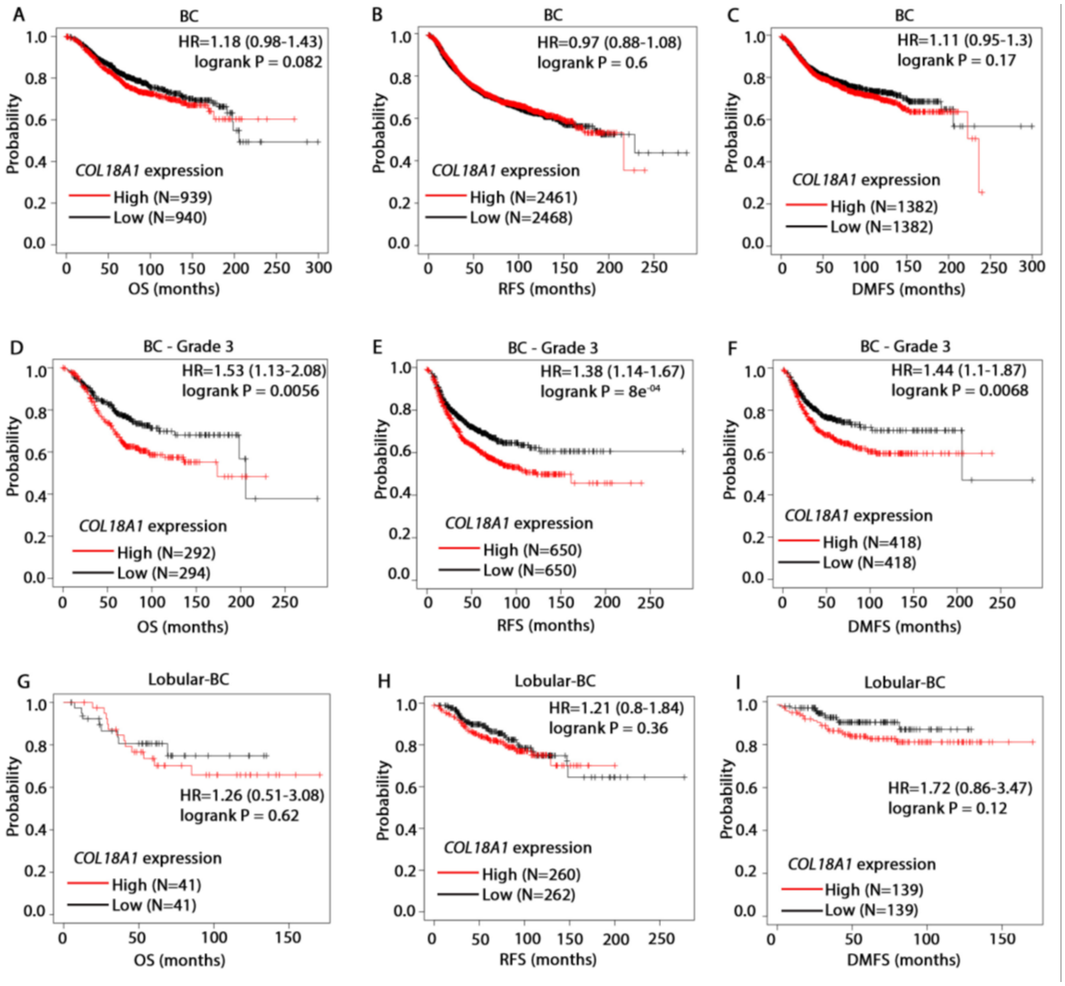

Supplemental Figure 4. *COL18A1* mRNA expression and breast cancer patient survival. Kaplan-Meier plots showing overall survival (OS), relapse-free survival (RFS) and distant metastasis-free survival (DMFS) of BC patients stratified by *COL*18*A1* mRNA expression levels (probe: 209082_s_at). **(A-C)** All available BC cases, **(D-F)** grade 3 BC cases and **(G-I)** lobular BCs (luminal A, estrogen receptor- and progesterone receptor-positive, HER2-negative). The open access gene expression data and patient survival information from TCGA, GEO and EGA compiled in a single database at www.kmplot.com (5) were used for the meta-analyses. High *COL*18*A1*, red line; low ColXVIII, black line. Hazard ratios (HR) and log-rank P values were computed using the median ColXVIII expression level as the cutoff. The number of patients for each analysis is indicated in the survival graphs. Panel E is shown also in Figure 1L.

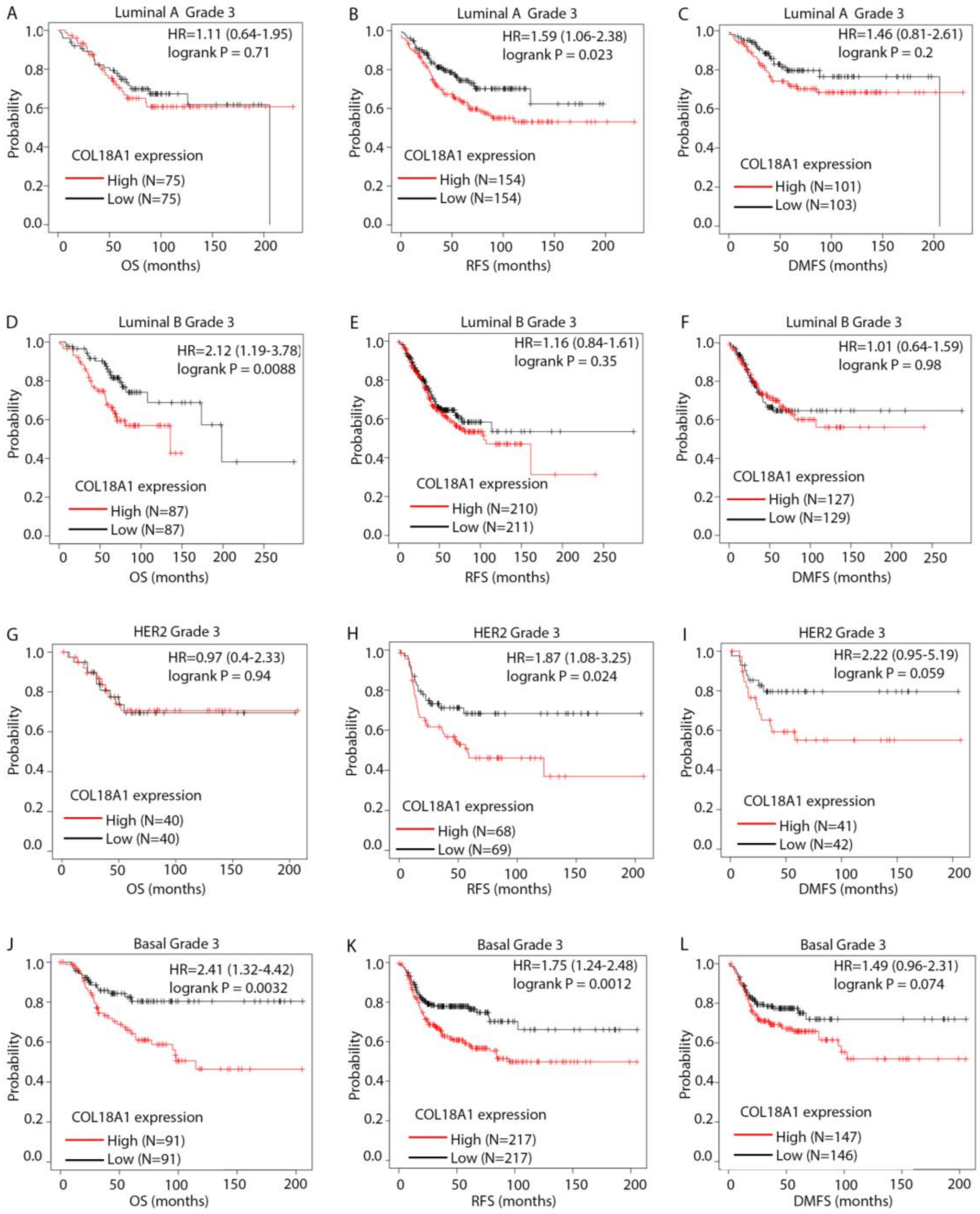

Supplemental Figure 5. *COL18A1* mRNA expression and patient survival in grade 3 breast cancers of different molecular subtypes. Kaplan-Meier plots showing overall survival (OS), relapse-free survival (RFS) and distant metastasis-free survival (DMFS) of BC patients stratified by *COL*18*A1* mRNA expression levels (probe: 209082_s_at). **(A-C)** Luminal A, **(D-F)** luminal B, **(G-I)** HER2 and **(J-L)** basal subtype of grade 3 BCs. The open access gene expression data and patient survival information from TCGA, GEO and EGA compiled in a single database at www.kmplot.com (5) were used for the meta-analyses. High *COL*18*A1*, red line; low *COL*18*A1*, black line. Hazard ratios (HR) and log-rank P values were computed using the median ColXVIII expression level as the cutoff. The number of patients for each analysis is indicated in the survival graphs. Panels B, H and K are shown also in Figure 1M–O.

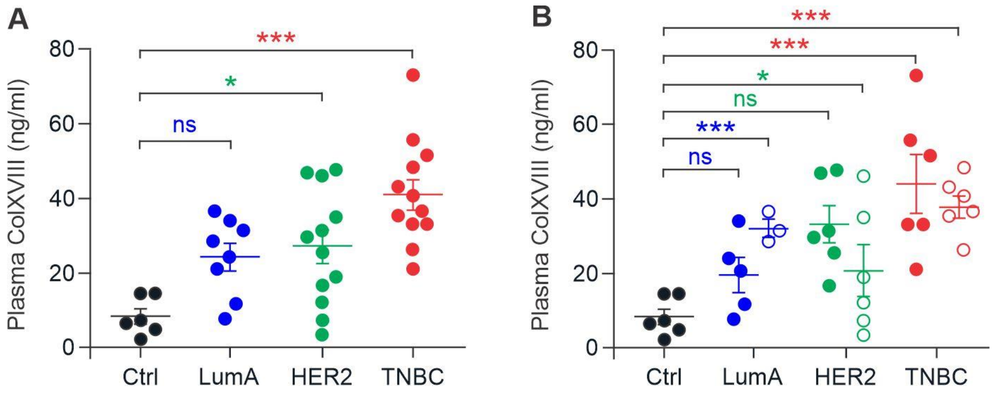

Supplemental Figure 6. ColXVIII expression in breast cancer patients' plasma. (**A**) Levels of circulating ColXVIII (ng/ml) in plasma samples of patients with luminal A (n=8), HER2 (n=12) and basal (n=12) subtypes of BC by comparison with healthy controls (n=6). (**B**) Levels of circulating ColXVIII (ng/ml) in plasma samples of BC patients classified according to the molecular subtype, and with and without lymph node metastasis. Filled circles, node-negative; open circles, node-positive. Numbers of plasma samples for luminal A type, node-negative n=5 and node-positive n=3; for HER2 type, node-negative n=6 and node-positive n=6; and for basal/TNBC type, node-negative n=6 and node-positive n=6. ColXVIII concentrations were measured using a monoclonal N-terminal TSP-1 domain-targeting anti-ColXVIII antibody (DB144-N2) (1) in an indirect ELISA assay (2 technical replicates). Student's 't' test was used in the statistical analysis. *, $p<0.05$. ***, $p<0.001$; and ns, not significant. Error bars represent s.e.m.

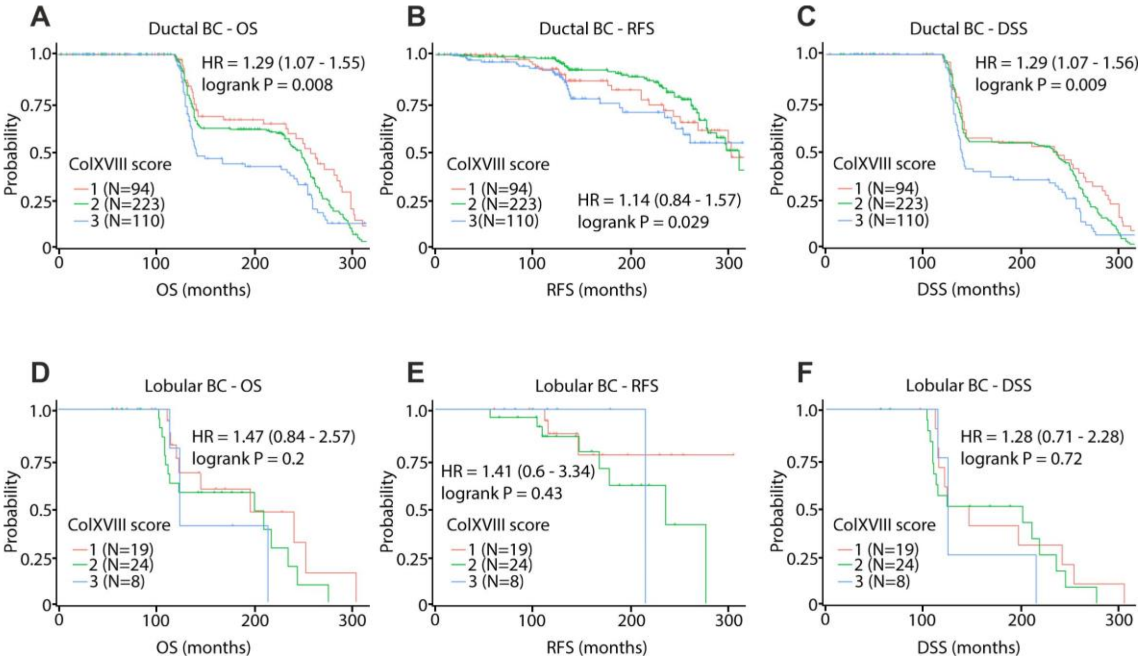

Supplemental Figure 7. Correlation between ColXVIII protein expression and survival of patients with invasive ductal breast cancer (IDC) or invasive lobular breast cancer (ILC). Cytoplasmic ColXVIII expression was scored by immunohistochemistry using the mAB DB144-N2 (1). Representative examples of ColXVIII scores 1 (weak), 2 (moderate) and 3 (strong) are shown in the Supplemental Figure 3. Kaplan-Meir plots showing overall survival (OS) (**A** and **D**), relapse-free survival (RFS) (**B** and **E**) and disease-specific survival (DSS) (**C** and **F**) for IDC (total N=427) and ILC (total N=51) patients, stratified by cytoplasmic ColXVIII protein expression levels. Survival analysis was done using Kendall rank correlation coefficient (Kendall Tau). Hazard ratio (HR) and log-rank P values were computed using the ColXVIII IHC scores as thresholds for stratification. The initial number of patients in each group (N) is indicated in the survival graphs.

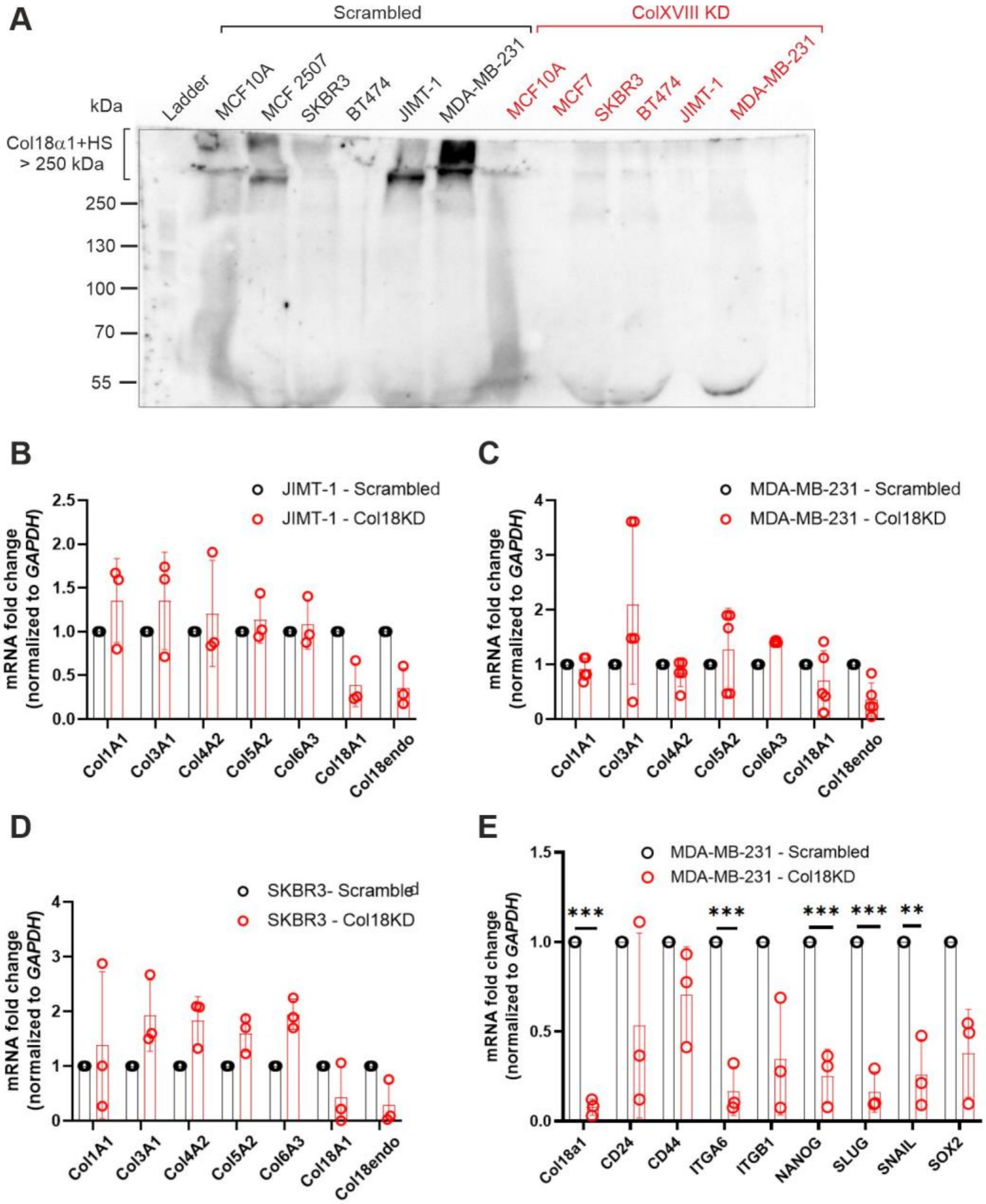

Supplemental Figure 8. ColXVIII knockdown (KD) in human breast cancer cell lines. (**A**) Representative Western blot showing that the secretion of high molecular weight, glycosylated ColXVIII into the conditioned culture medium is reduced in KD cells compared to corresponding control cells. pAB QH48.18 (2) was used to detect ColXVIII in Western blot. The experiment was repeated 3 times. HS, heparan sulphate. (**B**–**D**). A qRT-PCR analysis of relative expression levels of selected collagen α chain mRNA transcripts in human BC cell lines (scrambled vs. ColXVIII KD and normalized to *GAPDH*). Number of biological replicates for JIMT-1 cells (n=3) (B), MDA-MB-231 cells (n=5) (C), and SKBR3 cells (n=3). Two primer pairs were used for *COL*18*A1*, the OriGene primers and endostatin primers listed in Supplemental Table 5. (**E**) A qRT-PCR analysis of relative *COL18A1* expression levels and stem cell marker gene transcripts (scrambled vs. ColXVIII KD and normalized to *GAPDH*) in MDA-MB-231 cells (n=3 for all). In B–E, Student's 't' test was used in the statistical analysis. **, p<0.01; ***, p<0.001. Error bars represent s.d.

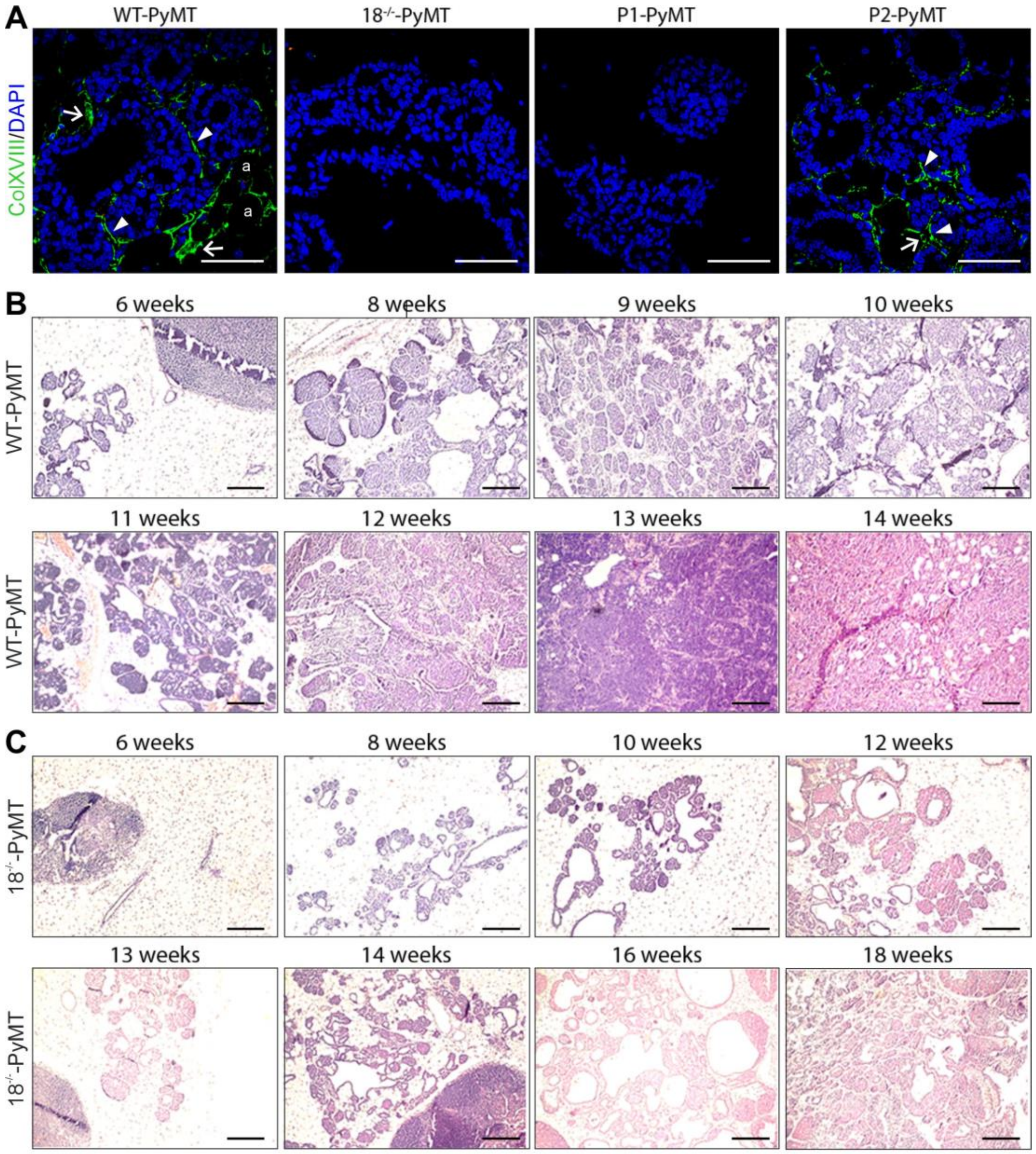

Supplemental Figure 9. Mammary carcinogenesis in MMTV-PyMT mice crossed with *Col18a1* mouse models. (**A**) Immunofluorescence staining of ColXVIII (green) in wild type MMTV-PyMT (WT-PyMT) mammary tumors, in $18^{-/-}$-PyMT tumors lacking all ColXVIII isoforms, in P1-PyMT tumors lacking the short ColXVIII and expressing long/medium ColXVIII, and in P2-PyMT tumors lacking the long/medium isoforms and expressing exclusively the short ColXVIII. The staining shows that the short ColXVIII is the prevalent isoform in mammary tumors. A polyclonal antibody targeting the N-terminal TSP-1 domain of mouse ColXVIII was used in the staining (6). The nuclei are counterstained with DAPI. At least 3 samples per genotype, at week 13, we stained. Scale bars, 100μm. Arrowhead, ductal basement membrane; arrow, blood vessel; a, adipocyte. (**B–C**) Representative images of hematoxylin-eosin staining of mammary tumors harvested from the *WT*-PyMT mice at the age of 6-14 weeks and from the $18^{-/-}$-PyMT mice at age 6-18 weeks. Mammary tumors transformed to carcinomas between weeks 10 and 13 in the *WT*-PyMT mice, and between weeks 16 and 18 in the $18^{-/-}$-PyMT mice. The tumors were classified according to Fluck and Schaffhausen (7). Scale bars, 200μM.

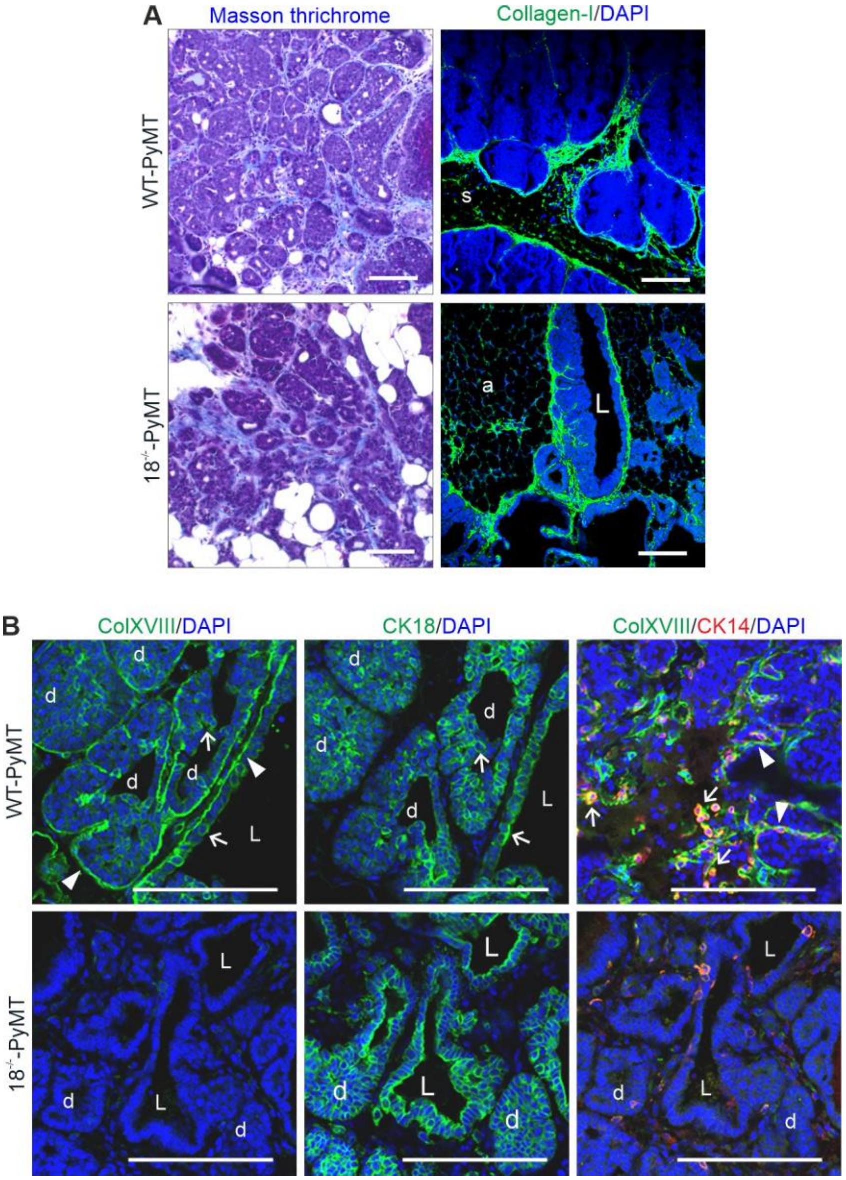

Supplemental Figure 10. Expression of fibrillar collagen, CK18 and CK14 in mouse mammary tumors. Representative images of immunostainings in WT-PyMT and $18^{-/-}$-PyMT tumor tissues at weeks 13. (**A**) Left, Masson's trichrome staining for collagen deposition (blue) in mammary tumors (n=5/genotype). Right, immunostaining for Collagen I (green), counterstained with DAPI (blue) (n=5/genotype). Scale bars, 100µm. s, stroma; a, adipocytes, L, lumen of a mammary duct. (**B**) Sequential mammary tumor sections stained for ColXVIII and CK18 (green). Double immunostaining for ColXVIII (green) and CK14 (red) (n=5/genotype). Scale bars, 100µm. d, duct; L, lumen of the duct; arrow, ColXVIII/CK18 or ColXVIII/CK14 double-positive cells. arrowhead, epithelial basement membrane.

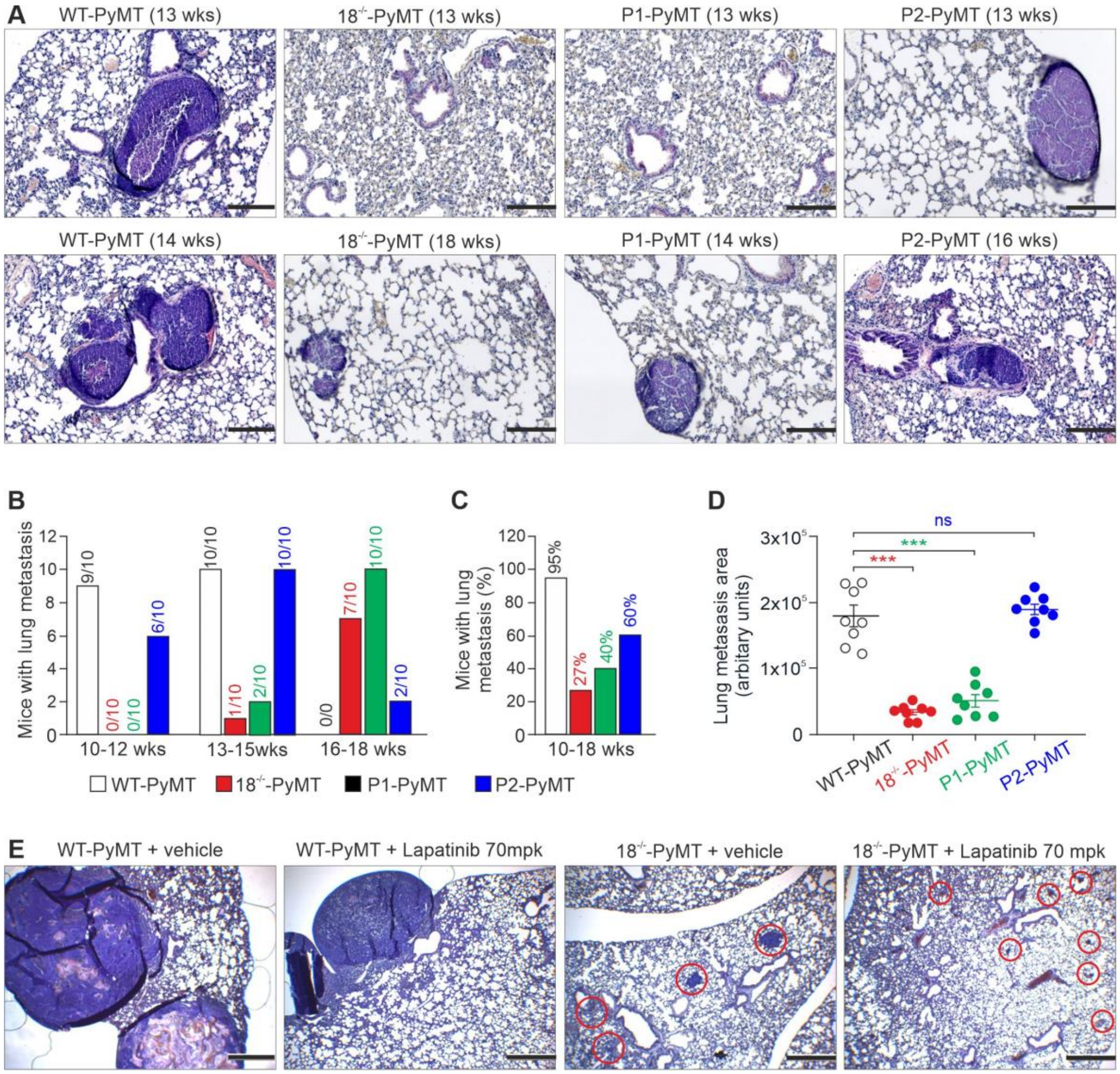

Supplemental Figure 11. Lung metastasis in MMTV-PyMT mice. (**A**) Hematoxylin-eosin-stained lung sections of WT-PyMT, $18^{-/-}$-PyMT, P1-PyMT and P2-PyMT mice at indicated age. (**B**) Numbers of mice with one or more lung metastases in the four PyMT mouse lines by age groups. The total number of examined mice of each group was ten, except for the WT-PyMT mice which could not be monitored beyond 14 weeks for ethical reasons. (**C**) Percentages of mice with lung metastasis in PyMT mouse lines at weeks 10-18. (**D**) ImageJ analysis of tumor areas in lung samples at weeks 13-18. One lung section per mouse was analyzed (n=8 for all genotypes). Student's 't' test was used for the statistical analysis. ***, $p<0.001$; ns, not significant. Error bars, s.e.m. (**E**) Lung metastasis in the WT-PyMT and $18^{-/-}$-PyMT mice treated with vehicle or ErbB inhibitor lapatinib (70 milligrams per kilo, mpk) for three weeks as depicted in the main Figure 8D. Hematoxylin-eosin-staining revealed macrometastases in the WT-PyMT lungs whereas in the $18^{-/-}$-PyMT lungs only micrometastases (red circles) were detected and these are smaller in the lapatinib-treated mice than in the vehicle-treated $18^{-/-}$-PyMT mice. (**A, E**) Scale bars, 200 μm.

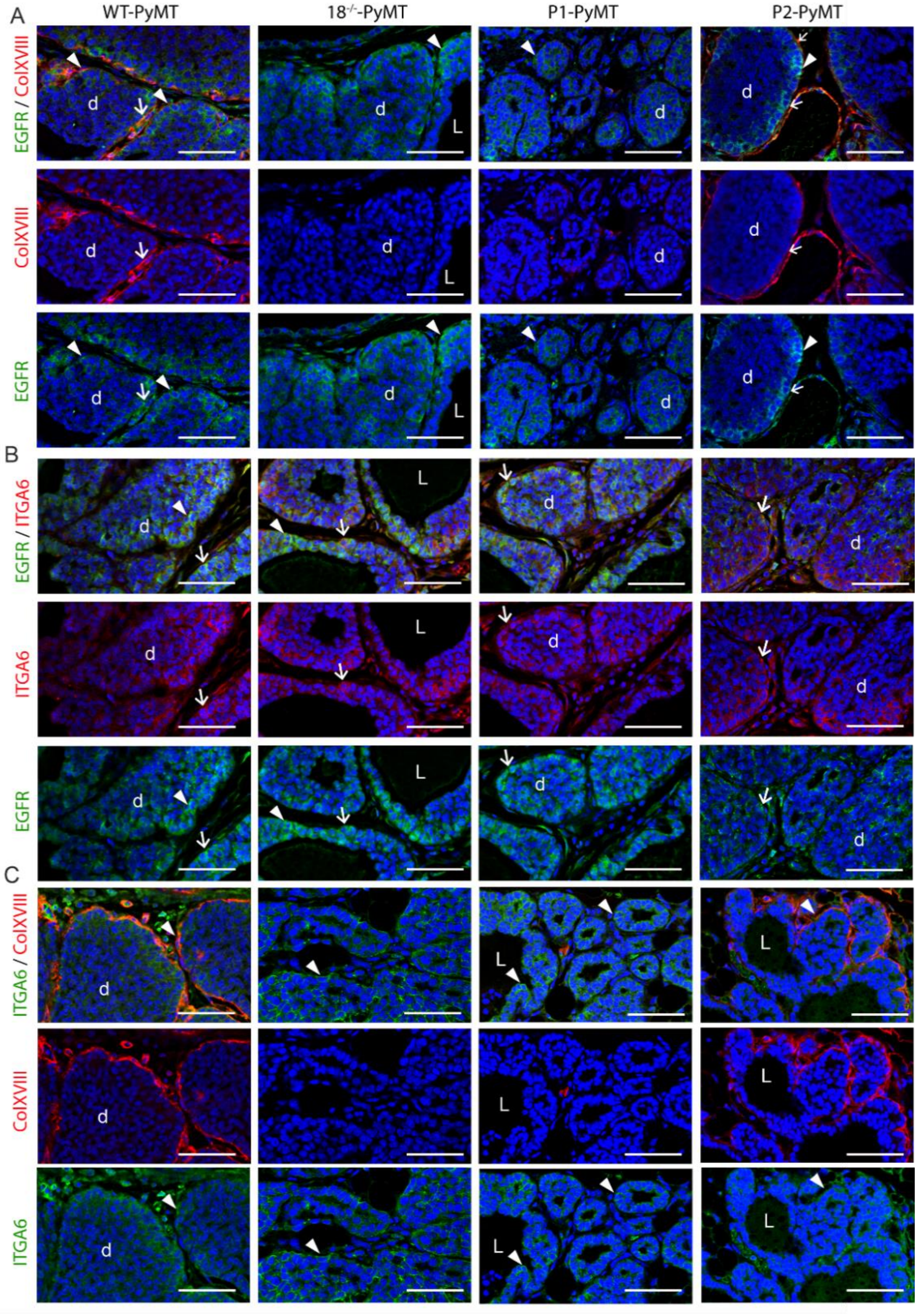

Supplemental Figure 12. Co-localization of ColXVIII, ErbBs and integrin α6 (ITGA6) in mouse mammary tumors. Representative images of immunostaining in WT-PyMT, $18^{-/-}$-PyMT, P1-PyMT and P2-PyMT mice mammary tumor tissues at week 13 (n=3/genotype. (**A**) ColXVIII (red) and EGFR (green), (**B**) ITGA6 (red) and EGFR (green), (**C**) ITGA6 (green) and ColXVIII (red). The nuclei are counterstained with DAPI. Arrow, co-localization points; arrowheads, membranous staining; d, duct, L, lumen. Scale bars, 100μm.

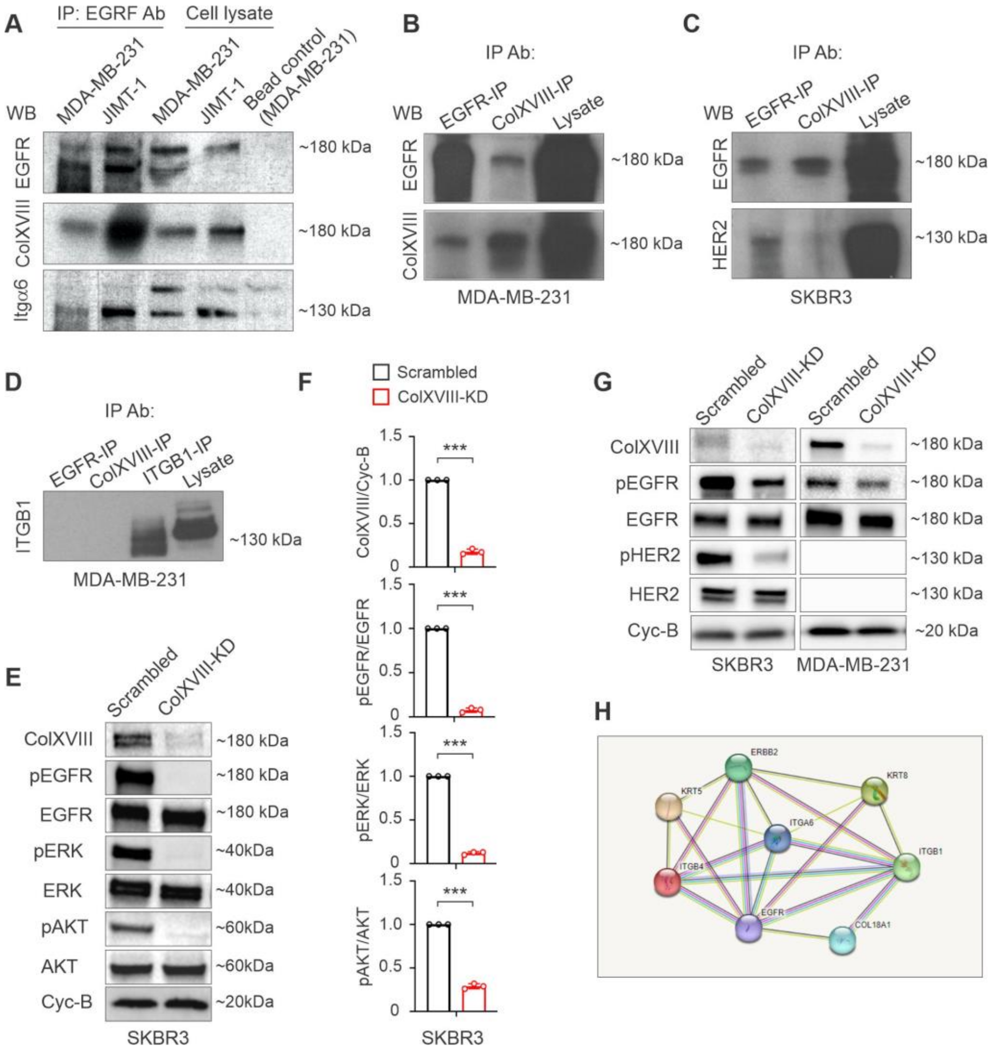

Supplemental Figure 13. Interactions between ColXVIII, ErbBs and integrins. (**A**) Co-immunoprecipitation (Co-IP) with an EGFR antibody (Abcam #52894) in HER2-negative MDA-MB-231 cells and HER2-positive JIMT-1 cells. Protein complexes were detected by Western blotting (WB) with anti-EGFR (Cell Signaling Technology, #4267), anti-ColXVIII (QH48.14)(2) and anti-α6 integrin (Abcam, #ab97760) antibodies. A goat anti-mouse IgG coated magnetic bead control is shown in the MDA-MB-231 cells. (**B**–**D**) Co-IP with EGFR (#52894) and ColXVIII (DB144-N2) antibodies in MDA-MB-231 (**B**) and SKBR3 (**C**) cell lysates, and with EGFR, ColXVIII (QH48.14) and integrin β1 (Millipore AB1952) antibodies in MDA-MB-231 lysates (**D**). In A–D, protein complexes were detected by WB with the EGFR (Cell Signaling Technology, #4267), ColXVIII (QH48.14), HER2 (Cell Signaling Technology, #4290) and integrin β1 (Millipore-AB1952) antibodies (Supplemental Table 2). (**E–G**) Representative immunoblots of EGFR and HER2 phosphorylation and downstream signaling mediators in Scrambled and ColXVIII KD SKBR3 (**E** and **G**) and MDA-MB-231 (**G**) cell lysates. Quantification of ColXVIII, and EGFR, ERK and AKT phosphorylation in SKBR3 cell lysates. Biological replicates in A–D, ≥ 5, an in E and G, ≥3. Student's 't' test was used for the statistical analysis. ***, p<0.001. Error bars represent s.d. (**H**) STRING network interaction analysis (https://string-db.org/) for selected proteins in epithelial cells. The colored lines indicate experimentally determined interactions (pink) and known interactions in curated database (light blue). Predicted interactions are indicated by gene neighborhood (green), gene fusions (red) and gene co-occurrence (blue). Other modes of interactions found by text mining (light green), co-expression (purple) and protein homology (grey).

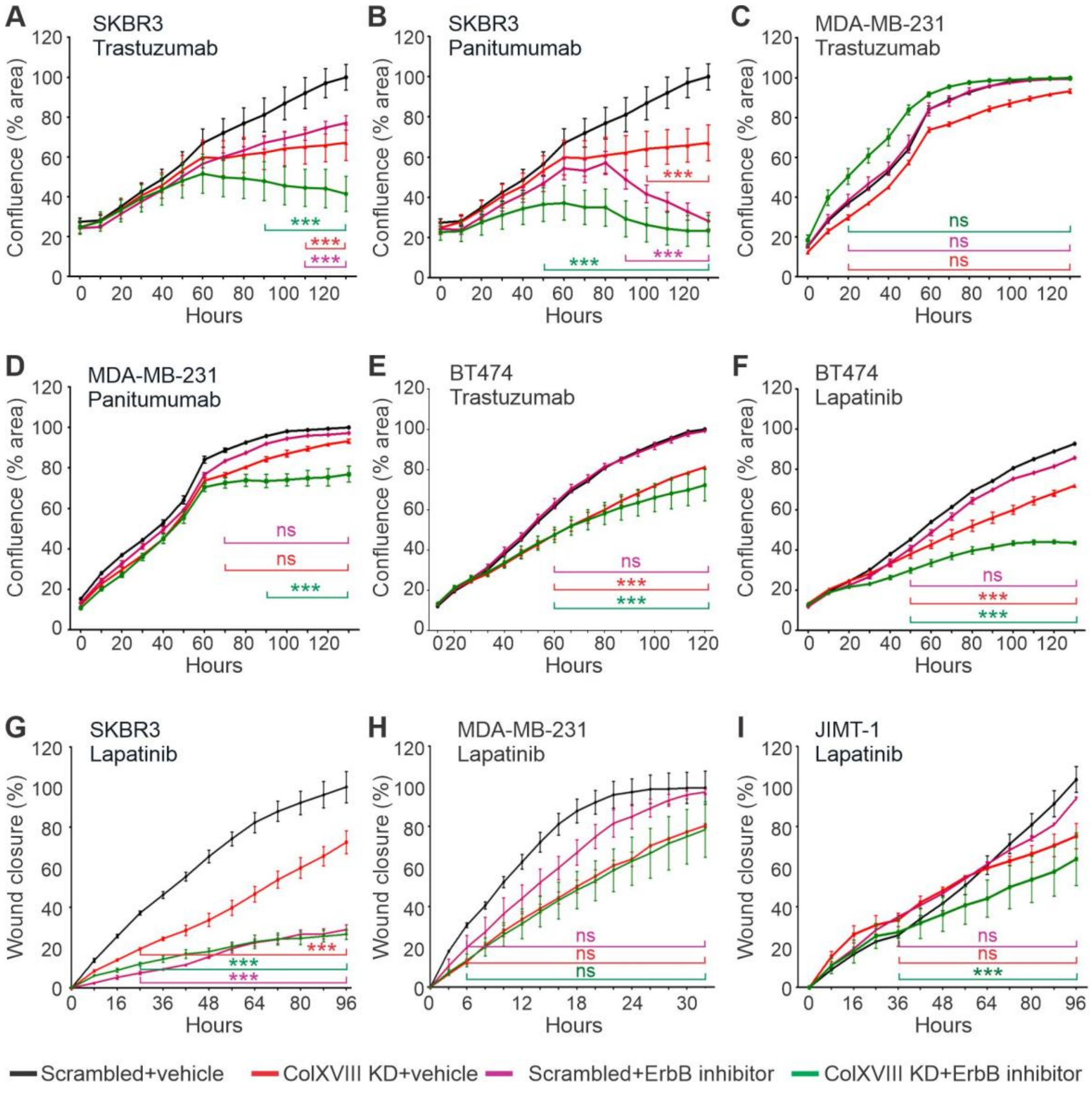

Supplemental Figure 14. Analysis of breast cancer cell proliferation and migration after ColXVIII knockdown and/or EGFR/ErbB-targeting drug treatment. (**A, B**) Proliferation of HER2-amplified SKBR3 cells after siRNA-based knockdown (KD) of *COL18A1* and combined with HER2-targeting mAB trastuzumab or the EGFR-targeting mAB panitumumab. (**C, D**) Proliferation of triple-negative MDA-MB-231 cells after *COL18A1* KD and combined with trastuzumab and panitumumab treatments. (**E, F**) Proliferation of BT474 cells (luminal B type, HER2-positive) after *COL18A1* KD and combined with trastuzumab and lapatinib treatments. (**G**–**I**) BC cells' migration after *COL18A1* KD and combined with lapatinib. Student's 't' test was used for the statistical analysis. ***, $p<0.001$; ns, not significant. Error bars represent s.e.m.

Supplemental Tables

Supplemental Table 1. Human breast cancer samples with histopathological and relapse data

	**Uppsala/Umeå cohort** (N=709)		**Oulu cohort** (N=21)	
	number	percentage	number	percentage
**Oestrogen (ER) receptor**				
Positive	614	86 %	11	52 %
Negative	73	11 %	10	48 %
NA	22	3 %		
**Progesterone (PR) receptor**				
Positive	496	70 %	10	48 %
Negative	193	27 %	11	52 %
NA	20	3 %		
**HER2 receptor**				
Positive	57	8 %	13	62 %
Negative	621	88 %	8	38 %
NA	30	4 %		
**Nuclear grade**				
1 (low)	54	7 %	2	10 %
2 (moderate)	445	63 %	4	19 %
3 (high)	196	28 %	15	71 %
NA	14	2 %		
**Ki67 status**				
Low	327	46 %	4	19 %
High	340	48 %	16	76 %
NA	42	6 %	1	5 %
**Invasive subtypes**				
Ductal	480	68 %	16	76 %
Lobular	54	7 %	5	24 %
Tubuloductal	149	21 %	NA	NA
Others	26	4 %	NA	NA
**Relapse**				
Yes	147	21 %	NA	NA
No	562	79 %	NA	NA

NA, data not available

Supplemental Table 2. Antibodies

Target/antibody	Catalog #	Manufacturer/Reference
Cy3-conjugate αSMA	C6198	Sigma
Cleaved Caspase-3	MAB835	R&D Systems
Collagen I (human)	PA5-95137	Invitrogen
Collagen I (human, mouse)	AB765P	Chemicon
Collagen IV (human)	600-401-106-0.5	Rockland
Collagen XVIII (human)	QH4818	In House (2)
Collagen XVIII (human)	DB144-N2	In House (1)
Collagen XVIII (mouse)	anti-NC11	In House (6)
Cyclophyllin B	SAB4200201	Sigma Aldrich
Cytokeratin 5 (mouse)	PRB-160P	Covance
Cytokeratin 8 (human)	ab71842	Abcam
Cytokeratin 8 (mouse)	ab109452	Abcam
Cytokeratin 14	ab7800	Abcam
Cytokeratin 18	ab52948	Abcam
EGFR (D38B1)	4267	Cell Signaling Technology
EGFR	ab2430	Abcam
EGFR	ab52894	Abcam
FITC-conjugated CD44	103005	Biosite
HER2/ErbB2 (D8F12)	4290	Cell Signaling Technology
Integrin alpha 6 (human)	ab97760	Abcam
Integrin alpha 6 (human)	555734	BD Biosciences
Integrin alpha 6 (mouse)	MAB1356	Chemicon
Integrin beta 1 (human)	AB1952	Sigma Aldrich
Integrin beta 1 (human)	SC9970	Santa Cruz
Ki67 (human)	M7248	Dako
Ki67 (mouse)	M7249	Dako
Lineage antibody cocktail (CD3, CD11b, CD45R/B220, Ter-119, Ly-6G/C)	558074	BD Biosciences
pan44/42 MAPK (Erk1/2) (137F5)	4695S	Cell Signaling Technology
phospho AKT (Tyr-326)	sc-109904	Santa Cruz
panAKT (c67e7)	4691	Cell Signaling Technology
PE -conjugated CD29	102207	Biolegend
PE/Cy7-conjugated CD24	101821	Biolegend
PE -conjugated CD49f	12-0495-82	eBiosciences
Phospho-Akt (Ser473) (D9E) XP	4060S	Cell Signaling Technology
Phospho-EGFR (Tyr1068) (D7A5)	3777	Cell Signaling Technology
Phospho-HER2/ErbB2 (Tyr1221/1222) (6B12)	2243	Cell Signaling Technology
Phospho-p38 MAPK (Thr180/Tyr182) (12F8)	4631S	Cell Signaling Technology

Phospho-p44/42 MAPK (Erk1/2) (Thr202/Tyr204)	4370S	Cell Signaling Technology
Phospho-PI3K p85 (Tyr458)/p55 (Tyr199)	4228	Cell Signaling Technology
PI3 Kinase Class III (D4E2)	3358	Cell Signaling Technology
PI3 Kinase p110α (C73F8)	4249	Cell Signaling Technology
PI3 Kinase p110γ (D55D5)	5405	Cell Signaling Technology
PI3 Kinase p110ß (C33D4)	3011	Cell Signaling Technology
PI3 Kinase p85 (19H8)	4257	Cell Signaling Technology
Tubulin (Beta)	MAB-3408	Millipore

Supplemental Table 3. Included as a separate Excel spreadsheet.

Supplemental Table 4. Human BC cell lines used in this study and their key characteristics.

Cell line	Subtype	ER	PR	HER2 (IHC)	EGFR (IHC)	Other data	References	Source
BT474	Luminal B, HER2	+	+	+++	+	*PIK3CA* mutated, resistant to trastuzumab and Lapatinib	(8, 9)	P.K., a gift
JIMT-1	HER2	–	–	+++	ND	*PIK3CA* mutated, resistant to trastuzumab	(9, 10)	DSMZ (# ACC589)
MCF10A	Basal, non-tumorigenic	–	–	–/+	++	–	(9, 11, 12)	Sigma Aldrich (# CLL1040)
MCF7	Luminal A	+	+	–/+	+	–	(8, 9, 11, 12)	ATCC (# HTB-22)
MDA-MB-231	Basal/TNBC	–	–	–	+++	*TP53, KRAS* and *BRAF* mutated	(9, 11, 12)	ATCC (# HTB-26)
SKBR3	HER2	–	–	+++	++	*TP53* mutated	(8, 9, 11, 12)	P.K., a gift
T47D	Luminal A	+	+	–	+	*PIK3CA* and *TP53* mutated	(8, 9, 11, 12)	P.K., a gift

N.D., no previous data available. P.K., Professor Peppi Karppinen, University of Oulu.

Supplemental Table 5. qRT-PCR Primers

Gene Symbol	Gene Name	Primers	Sequences (5' → 3')
COL1A1	Collagen type I, alpha 1 chain (OriGene)	Forward	GATTCCCTGGACCTAAAGGTGC
		Reverse	AGCCTCTCCATCTTTGCCAGCA
COL3A1	Collagen type III, alpha 1 chain (OriGene)	Forward	TGGTCTGCAAGGAATGCCTGGA
		Reverse	TCTTTCCCTGGGACACCATCAG
COL4A2	Collagen type IV, alpha 2 chain (OriGene)	Forward	GGATAACAGGCGTGACTGGAGT
		Reverse	CTTTGCCACCAGGCAGTCCAAT
COL5A2	Collagen type V, alpha 2 chain (OriGene)	Forward	CAGGCTCCATAGGAATCAGAGG
		Reverse	CCAGCATTTCCTGCTTCTCCAG
COL6A3	Collagen type VI, alpha 3 chain (OriGene)	Forward	CCTGGTGTAACTGATGCTGCCA
		Reverse	AAGATGGCGTCCACCTTGGACT
*COL*18*A1*	Collagen, type XVIII, alpha 1 chain (OriGene, all isoforms)	Forward	GGAGAGATTGGCTTTCCTGGAC
		Reverse	CCTCATGCCAAATCCAAGGCTG
*COL*18*A1*	Collagen, type XVIII, alpha 1 chain (C-terminal, endostatin, all isoforms)	Forward	TGCCCATCGTCAACCTCAAG
		Reverse	CAGAGCCTGAGAACAGAGCC
*COL*18*A1*	Collagen, type XVIII, alpha 1 chain (N-terminal, Short isoform)	Forward	CTCCTGGACGTGCTCGC
		Reverse	TCATCCGTCTGGGTGACCT
*COL*18*A1*	Collagen, type XVIII, alpha 1 chain (N-terminal, Medium isoform)	Forward	GCCGTGGCATTCCTAGCTC
		Reverse	CTGATGCGCTCTGAAGATGGT
*COL*18*A1*	Collagen, type XVIII, alpha 1 chain (N-terminal, Long isoform)	Forward	AGCTTCTCTCTCCTCCTTGCT
		Reverse	GCGAGAGTCCTTGGCTGTCT
*Col*18*a1*	Collagen, type XVIII, alpha 1 chain (C-terminal, endostatin, all isoforms)	Forward	GTGCCCATCGTCAACCTGAA
		Reverse	GACATCTCTGCCGTCAAAAGAA
*Col*18*a1*	Collagen, type XVIII, alpha 1 chain (N-terminal, Short isoform)	Forward	GGATGTGCTCACCAGTTTGG
		Reverse	GTCATCGATTTGTGAGATCTTC
*Col*18*a1*	Collagen, type XVIII, alpha 1 chain (N-terminal, Medium isoform	Forward	ACCTCCAGGCACCACTGGGA
		Reverse	GCCCGACGTGAGGGTCATCG
*Col*18*a1*	Collagen, type XVIII, alpha 1 chain (N-terminal, Long isoform)	Forward	AGGAGGACGGGTACTGTGTG
		Reverse	TGAGGGTCATCGATTTGTGA
CD24	CD24 molecule	Forward	CTCCTACCCACGCAGATTTATTC
		Reverse	AGAGTGAGACCACGAAGAGAC
CD44	CD44 molecule	Forward	CTGCCGCTTTGCAGGTGTA
		Reverse	CATTGTGGGCAAGGTGCTATT
CDH1	Cadherin 1	Forward	ATTTTTCCCTCGACACCCGAT
		Reverse	TCCCAGGCGTAGACCAAGA
GAPDH	glyceraldehyde-3-phosphate dehydrogenase	Forward	ACAACTTTGGTATCGTGGAAGG
		Reverse	GCCATCACGCCACAGTTTC

ITGA6	Integrin alpha 6	Forward	GGCGGTGTTATGTCCTGAGTC
		Reverse	AATCGCCCATCACAAAAGCTC
ITGB1	Integrin beta 1	Forward	GTAACCAACCGTAGCAAAGGA
		Reverse	TCCCCTGATCTTAATCGCAAAAC
KLF4	Kruppel like factor 4	Forward	CAGCTTCACCTATCCGATCCG
		Reverse	GACTCCCTGCCATAGAGGAGG
NANOG	Nanog homeobox	Forward	CCCCAGCCTTTACTCTTCCTA
		Reverse	CCAGGTTGAATTGTTCCAGGTC
SNAIL1	Snail family zinc finger 1	Forward	GTGTCTCCCAGAATATT
		Reverse	GTTTGAAATATAAATACCAGTGT
SOX2	SRY-box 2	Forward	TACAGCATGTCCTACTCGCAG
		Reverse	GAGGAAGAGGTAACCACAGGG
SLUG	Snail family transcriptional repressor 2	Forward	GCTGATGGCTAGATTGAG
		Reverse	ATCCTATTACAGACTCTATTACAA
TWIST2	Twist basic helix-loop-helix transcription factor 2	Forward	TTCTCCGTGATTGCTTGG
		Reverse	AGGATACACAGCCACACT
VIM	Vimentin	Forward	AGTCCACTGAGTACCGGAGAC
		Reverse	CATTTCACGCATCTGGCGTTC

Supplemental materials and Methods

Human breast cancer samples and ethical permits

Formalin-fixed paraffin-embedded (FFPE) human breast cancer (BC) samples representing different subtypes were received from two patient cohorts, at Oulu University Hospital, Finland (N=21), and Uppsala/ Umeå University Hospital, Sweden (N=709) (Supplemental Table 1), the latter assembled in a tissue microarray (TMA) in which each sample was represented in duplicate. The plasma samples from BC patients (N=8-12 per molecular subtype) were from the Umeå University Hospital cohort. The control plasma samples were collected from 6 healthy women volunteers above 45 years of age. All human samples and clinical data were anonymized and labelled with a research code for blinded histopathological (Supplemental Table 3C) and plasma analyses. Only authorized personnel of the Oulu, Uppsala and Umeå University Hospitals had access to the personal and clinical data. Informed consent for data use was obtained from all the patients and volunteers prior to participation. The Ethical Committee at the Medical Faculty of Umeå University (Dnr 09-175M), the Regional Ethics Review Board in Uppsala (Dnr 2005:118) and the Ethical Committee of the Northern Ostrobothnia Health Care District (Dnr 88/2000 and amendments Dnr 194/2013 and Dnr 100/2016) granted approvals for the use of these samples.

Immunohistochemistry of human BC samples and evaluation of ColXVIII and EGFR expression

Immunohistochemical (IHC) staining of 5 μm thick FFPE breast tumor sections was performed using the Dako REAL™ EnVision™ detection system (Dako, #K5007). The primary antibodies used were an in-house mouse monoclonal antibody (mAB) DB144-N2 which targets the N-terminal portion of the molecule of human ColXVIII (1), dilution 1:150; an in-house rabbit polyclonal antibody (pAB) QH48.18 which targets the same N-terminal region of human ColXVIII (2), dilution 1:1000; rabbit mAB for human EGFR (Cell Signaling Technology, #4267), dilution 1:50; rabbit mAB for human HER2 (Cell Signaling Technology, #4290), dilution 1:50; and mouse mAB for human Ki67 (Dako, #M7248), dilution 1:50 (Supplemental Table 2). Specificity of the custom-made DB144-N2 and QH48.18 antibodies was inspected by Western blot with recombinant ColXVIII fragments (Supplemental Figure 1), and previously in (1) and (2), respectively. Images were captured using a Leica DM LB2 light microscope or automatic scanning using a Zeiss Axio Imager M2. ColXVIII and EGFR staining were evaluated by three independent observers. ColXVIII expression was scored based on the intracellular staining intensity as follows: 0, no cytoplasmic staining in tumor cells or weak cytoplasmic staining in ≤10% of tumor cells; 1+, weak or moderate cytoplasmic staining in ≥10% of tumor cells; 2+, moderate cytoplasmic staining in ≥50% of tumor cells; 3+, strong cytoplasmic staining in ≥50% of tumor cells. In all specimens, vascular basement membranes (BM) were positive for ColXVIII, and the BM/myoepithelium presented either negative, fragmented or continuous positive signals for ColXVIII, depending on tumor grade. EGFR expression was scored as follows: 0, no membranous staining or weak cytoplasmic staining in tumor cells; 1+, weak membranous staining in ≥25% of the tumor cells; 2+, moderate membranous staining in ≥50% of the tumor cells; 3+, strong membranous staining in ≥50% of the tumor cells (Supplemental Figure 3). Cytoplasmic EGFR was observed in all samples, ranging from weak to strong and correlating with the intensity of membranous EGFR staining. Consistency of the scoring was tested using linear regression or Pearson correlation, and

non-matching scores were removed from the final analysis. For Uppsala/Umeå cohort samples the nuclear grade, HER2 and Ki67 expression had been scored previously by subspecialized breast pathologists at Umeå University Hospital (13) and at Oulu University Hospital. Representative cases of BC showing cytoplasmic ColXVIII and membrane EGFR expression are presented in Supplemental Figure 3.

Kaplan-Meier survival analysis

Kaplan-Meier survival analyses were performed by accessing BC patients' data for *COL*18*A1* gene expression (Affymetrix *COL18A1* probe: 209082_s_at 'TCAGCCAGCACTTGTCCAGCTGAGC' targeting the endostatin domain) using the website (5). This database provides a compilation of data from several open databases, including the Cancer Genome Atlas (TCGA), the Gene Expression Omnibus (GEO and the European Genome-phenome Archive (EGA). The association of *COL18A1* mRNA expression levels with overall survival (OS), relapse free survival (RFS) and distant metastasis-free survival (DMFS) was analyzed either in unclassified form or classified according to the differentiation grade or molecular subtype. Hazard ratios and log-rank P value were computed using the median ColXVIII expression level as the cutoff. In the Uppsala/Umeå cohort (n=630), the association of cytoplasmic ColXVIII protein expression levels with OS, RFS and disease-specific survival (DSS) was analyzed using R and ColXVIII IHC scores as thresholds for stratification

Enzyme-linked immunosorbent assay (ELISA) for plasma samples

Indirect ELISA was carried out to detect the N-terminal ColXVIII fragments in plasma samples collected from BC patients and healthy controls. 100µl of plasma samples, diluted 1:10 in 100 mM carbonate/bicarbonate coating buffer, pH 9.6, were pipetted in duplicate onto a 96-well ELISA microplate (Nalgene Nunc). Linear dilutions (1-300 ng/ml in coating buffer) of the standard, the recombinant N-terminal NC11 fragment of ColXVIII (3), were added in duplicate. The plates were incubated overnight at 4°C with gentle shaking and washed thrice with PBS-0.05% Tween (PBST). The remaining protein binding sites in the coated wells were blocked by adding 200µl of blocking buffer (1% BSA in PBST) and incubating at +37°C for 1h. After washing the wells thrice with PBST, 100µl of monoclonal DB144-N2 antibody against the TSP-1-like domain of ColXVIII (1), diluted 1:1000 in blocking buffer, was added and incubation was continued for 1 h at +37 °C. After washing thrice with PBST, an anti-mouse HRP-conjugated secondary antibody at a dilution of 1:10 000 was added to each well and incubated for 1 hr at +37°C. Control wells without primary or secondary antibodies were included in the assay. After washing thrice with PBST, 100µl of freshly constituted 3,3',5,5'-tetramethylbenzidine (TMB) substrate was added and the reaction was allowed to proceed at room temperature without shaking. The colour reaction was stopped after 15-20 min by adding an equal volume of 1M H_2SO_4. The absorbance was read at a wavelength of 450 nm and a correction wavelength of 650 nm using a Tecan Infinite M1000 PRO microplate reader. The concentration of ColXVIII in the plasma samples was determined from the standard curve and the results were analysed using Graph-pad Prism statistical software and represented in graphs.

Human breast cancer cell lines

Human BC cell lines used in this study and their key characteristics are listed in Supplemental Table 4. MDA-MB-231 (HTB-26) and MCF7 (HTB-22) cells were purchased from the American

Type Culture collection, MCF10A (CLL1040) cells from Sigma-Aldrich, and JIMT-1 (ACC589) cells from DSMZ, Germany. SKBR3, BT474 and T47D cells were a kind gift from Professor Peppi Koivunen, University of Oulu. Cell lines other than MCF10A were cultured in Dulbecco's Modified Eagle's Medium (DMEM) with high glucose and supplemented with 10% fetal bovine serum (FBS), 1% penicillin/streptomycin and 1% GlutaMax (all from Gibco). MCF10A cells were cultured in DMEM:F12 medium containing 5% horse serum (Invitrogen 16050-122) FBS, 10 μg/ml of insulin (Sigma-Aldrich #I9278), 20 ng/ml of EGF (Sigma-Aldrich #E9644), 100 ng/ml of cholera toxin (Sigma-Aldrich #C8052), 0.5 μg/ml of hydrocortisone (Sigma-Aldrich #H0888) and 1% penicillin/streptomycin. All the cell lines were maintained at 37°C with 5% ambient CO_2. and found negative in routine mycoplasma infection tests.

siRNA mediated *COL18A1* gene knockdown

Transfections were performed by first incubating 500µl of Opti-MEM media (Gibco) with 7.5µl of lipofectamine (RNAiMAX, Invitrogen) in each well of the 6-well plate for 5 min at RT. After that 10nM of siRNAs were added, mixed gently, and incubated for an additional 15 min at RT. BC cells were added to the transfection mixture at a density of 2.5×10^5 cells per well. For an efficient knockdown of ColXVIII, a mixture of two commercially available siRNAs targeting two distinct areas in the *COL*18*A1* mRNA transcript were used, namely SASI_Hs01_00161793 (Sigma-Aldrich) for the 5′- GCATCTTCTCCTTTGACGGCAAGGA-3′ sequence in the N-terminal TSP-1 domain, and Hs_*COL18A1*_3 (Qiagen) for the sequence 5′-CCCGGGATGAACGGATTGAAA-3′ in the C-terminal endostatin domain. 10nM of scrambled siRNA with a random nucleotide sequence (AllStars Negative Control siRNA, Qiagen) was transfected into cells in the same way and used as a negative control. Knockdown efficiency was estimated with qRT-PCR for the *COL18A1* mRNA transcripts and Western blot for the ColXVIII protein. Transfection efficiency was ensured with Allstar Hs Cell Death Control siRNA (Qiagen), which kills the cells that have become transfected. The transfected cells were further seeded at a density of 5×10^3 or $3–4\times10^4$ cells/well in a 96-well plate, depending on the protocol.

Cell proliferation and migration assays

For the cell proliferation and migration analyses, live cell phase contrast imaging was performed with IncuCyte ZOOM (Essen Biosciences). For the proliferation assays 5×10^3 cells/well were plated onto 96-well cell culture plates and cell proliferation curves were drawn from confluence measurements acquired during round-the-clock kinetic imaging at 2-hour intervals. To study the effects of specific non-collagenous (NC) domains of ColXVIII on cell proliferation, recombinant TSP-1(4), endostatin(14) and the NC11 fragment of the long ColXVIII(3) were produced in mammalian cells and added to cells at final concentrations of 500 ng/ml. In the cell migration assays $3-5\times10^4$ cells/well were plated onto 96-well ImageLock plates (Essen Bioscience). At 90–100% confluence the plates were scratched with a 96-well wound maker (Essen Bioscience) and cell migration was detected with IncuCyte by recording one image per well every two hours for 30-96 hours. Cell proliferation was quantified by determining the confluence of the surface area of the 2D culture over time and cell migration by monitoring wound closure using the Incucyte zoom software. Statistical analyses were performed with Graph-pad Prism software.

Mouse models and ethical permits

Previously generated *Col18a1*$^{-/-}$ knockout mice (*Col18a1*^{tm1Hms}, MGI:2179134) lacking all three ColXVIII isoforms (15), *Col18a1*^{P1} mice (*Col18a1*^{tm1Pih}, MGI:4948124) lacking the short ColXVIII isoform and *Col18a1*^{P2} mice (*Col18a1*^{tm2Pih}, MGI:4948125) lacking the medium/long isoforms (3) were used. All three ColXVIII mouse lines were backcrossed into the FVB/N (Harlan, the Netherlands) background for at least 8 generations. These mouse lines were then intercrossed with the transgenic MMTV–PyMT mouse line [FVB/N-Tg (MMTV-PyMT)634Mul/J, MGI:2161653] (16) (Jackson Laboratory, USA) for mammary carcinogenesis studies. All the animal experiments were approved by the Finnish National Animal Experiment Board (permissions ESAVI/6105/04.10.07/2015, ESAVI/1188/04.10.07/2016 and ESAVI-2936-04.10.07/2016) and conducted in the University of Oulu Laboratory Animal Centre.

MMTV-PyMT mammary carcinogenesis studies

The MMTV-PyMT mouse lines were maintained by breeding the PyMT transgene-positive male mice, either wild type (PyMT) or various *Col18a1* knockout males (i.e. *Col18a1*$^{-/-}$, *Col18a1*$^{P1/P1}$ and *Col18a1*$^{P2/P2}$ males) with PyMT-negative wild type FVB/N females or *PyM*T-negative knockout females to obtain female mice that were hemizygous for the PyMT transgene for use in the mammary carcinogenesis experiments. The resulting mouse lines are referred to in this paper as WT-PyMT (controls) and 18$^{-/-}$-PyMT, P1-PyMT and P2-PyMT. The mice were group-housed in corncob bedding with enrichments, given purified water ad libitum and irradiated standard rodent chow, and maintained on a 12:12-h light: dark cycle. All the data reported here refer to female mice monitored at specific time intervals starting at the age of five weeks and continuing up to 14 weeks of age for the WT-PyMT mice and up to 18 weeks for the 18$^{-/-}$-PyMT mice. The mice were sacrificed with CO_2 inhalation and cervical dislocation at predetermined time points, or upon reaching a humane endpoint criterion (size of a single mammary gland over 1 cm in diameter, high total tumor burden, loss of weight, reduced mobility, hunched posture, low activity, appetite loss, or closed eyes). The mammary glands were then dissected and weighed. For further analyses, the mammary glands were fixed with 4% paraformaldehyde (PFA) in phosphate-buffered saline (PBS), pH 7.4, and embedded in paraffin, or directly embedded in O.C.T. cryo-compound (Tissue-Tek OCT, Sakura) and stored at -80°C, or else snap-frozen in liquid nitrogen for protein and RNA analyses. Survival analysis for the WT-PyMT (n=38), 18$^{-/-}$-PyMT (n=31), P1-PyMT (n=28) and P2-PyMT (n=33) mice was performed by following the subjects until the humane end point (code=1) or ruled out for noncompliance (code=0) and calculating the log rank (Mantel–Cox) test using Graph-pad Prism software.

Mouse tissue stainings

The tissue morphology of the mammary gland tumors was assessed in hematoxylin-eosin-stained 5 μm sections from the PFA-fixed tumor samples. For immunofluorescence staining, the PFA-fixed samples were deparaffinized for 3 min in xylene and then rehydrated with decreasing ethanol concentrations of 95, 85 and 70% and finally PBS, twice for 3 min in each solution. Heat-mediated antigen retrieval was accomplished by boiling the sections in 0.1M citrate buffer, pH 6.0, for 20 min. The sections were then blocked with 1% BSA + 0.3M glycine in a 1×Tris-buffered saline, 0.1% Tween-20, pH 7.5 (TBST) buffer followed by overnight incubation at +4°C with polyclonal antibodies) for mouse ColXVIII (anti-NC11) (6), Collagen I (AB765P, Chemicon), Ki67 (Dako, #7249), cleaved caspase 3 (R&D systems, #MAB835), cytokeratin (CK) 8 (ab109452, Abcam),

CK18 (ab52948, Abcam), CK5 (CK5, Covance #PRB-160P), CK14 (ab7800, Abcam), integrin α6 (Abcam, #181551) and integrin β1 (Millipore #MAB1997) (Supplemental Table 2). After incubation with the primary antibodies, the slides were washed thrice for 5 min in PBS, followed by incubation with respective fluorochrome-conjugated Alexa Fluor 488- or Cy3-conjugated secondary antibodies and DAPI (Sigma-Aldrich) for 1 hour at RT. After three 5-min washes with PBS, the samples were mounted in Immu-Mount medium (Thermo Fisher Scientific). In the case of Cy3-conjugated αSMA (Sigma-Aldrich, #C6198) the secondary antibody was omitted. 7-μm cryosections on poly-L-lysine coated slides were fixed for 20 min in methanol at –20°C, air-dried, washed for 5 min in PBS and then processed according to the above protocols. Images were acquired using Zeiss LSM 780 and LSM 700 confocal microscopes, and image analyses were performed using Fiji ImageJ or Zen 2011 software. For Masson's thrichrome staining, the sections were treated with a nuclear stain (Celestine blue Harris hematoxylin, followed by treatments with acid fuchsin and phosphomolybdic acid, and, finally, with methyl methyl blue stain (all reagents from Sigma-Aldrich). Morphometric analyses for CK5, CK8, CK14 and Ki67 stainings were performed by capturing the images with a 20× objective using a Zeiss LSM 700/780 confocal microscope and counting positive cells with the Fiji ImageJ software. The count of positive cells was reported as the average of 4 areas of the tumor tissue.

Carmine Alum staining of mouse mammary glands.

Carmine Alum staining was performed as previously described (17). Briefly, whole mammary glands were mounted on a microscope slide, fixed in 4% PFA in PBS, pH 7.4, overnight, and transferred to 70% ethanol. The mounted slides were then rinsed in water and stained in a filtered solution of 0.2% Carmine (Sigma-Aldrich) and 0.5% aluminum potassium sulfate dodecahydrate (Sigma-Aldrich). The samples were dehydrated through sequential 70, 95 and 100% ethanol washes (20 min in each) and stored in a methyl salicylate solution.

Orthotopic allograft transplantation assay

The allograft transplantation assay was performed as a reciprocal transplantation of WT-PyMT and $18^{-/-}$-PyMT tumor cells into mammary fat pads of *WT* and *Col18a1*$^{-/-}$ FVB mice at 5 weeks of age. The WT-PyMT and $18^{-/-}$-PyMT donor mice were euthanized and sterilized with 70% ethanol. The mammary glands were exercised and placed onto a tissue-culture plate containing DMEM: F12 medium (ATCC cat no. 30-2020) and extra fat was carefully removed, and the tumors minced with scalpel blades inside laminar hoods. The minced tissues were transferred to 0.3% collagenase (LS004176, Worthington) and incubated for one hour at +37°C. The cell suspension was filtered through a 70 μm cell strainer (BD Biosciences), centrifuged and cell pellets isolated, suspended, and passed again through a 70 μm cell strainer. The tumor cell suspension was reconstituted at 5×10^6 cells/ml in PBS and 50μl of this suspension (2.5×10^5 tumor cells) was injected into abdominal mammary fat pads. The experimental groups were the following: Group-1, *WT*-FVB mice receiving WT-PyMT tumor cells; Group-2, *WT*-FVB mice receiving $18^{-/-}$-PyMT tumor cells; Group-3, *Col18a1*$^{-/-}$ FVB mice receivied WT-PyMT tumor cells; and Group-4, *Col18a1*$^{-/-}$ FVB mice receiving $18^{-/-}$-PyMT tumor cells. The tumors were measured with a caliper twice per week, and their volumes calculated using the formula: length × width^2 × 0.52. The experiment was terminated at week 10, when the WT-PyMT tumors reached an average volume of 600 mm^3, or at week 17 in the case of the $18^{-/-}$-PyMT tumors, when the largest tumors reached an average of 400 mm^3.

RNA isolation and qRT-PCR

RNA from human cell lines and mouse tissue samples was isolated using the Qiagen kit (RNeasy mini), and cDNA was produced with the iScript cDNA synthesis kit (Bio-Rad). Quantitative real-time PCR (qRT-PCR) was performed with SYBR Green Supermix (Bio-Rad). All the assays were performed in duplicate using a CFX96 Real-Time System (Bio-Rad). The values for all the samples were normalized to *Gapdh* (mouse) or *GAPDH* (human), and the relative fold change ($2^{\Delta\Delta}$Cq) was calculated using CFX Manager Software (Bio-Rad). The qRT-PCR primers used here are listed in Supplemental Table 4.

Western Blotting

To prepare the human BC cell lysates, the culture medium was aspirated, and cells were washed once in ice-cold PBS, after which NETN-300 buffer (20 mM Tris-HCl, pH 7.5, 300 mM NaCl, 1 mM EDTA, 0.5% NP-40) supplemented with 1× complete mini EDTA protease inhibitor (Roche Diagnostics) was pipetted directly onto the plate before collecting the cells using a cell scraper. Cell lines and tumor tissue lysates were cleared by centrifugation at 20,000 × g for 20 min at 4°C and the protein concentration was measured using a DirectDetect IR spectrometer (Merck Millipore). Secreted proteins in conditioned media (CM) were precipitated with acetone (1 part CM + 4 parts ice-cold acetone v/v) by incubating the mixture overnight at -20°C. The solution was centrifuged for 10 min at 15000 x g and supernatant was discarded. The lysates/pellets were suspended in 2.5× Laemmli buffer, separated using SDS-PAGE and blotted onto PVDF membranes. The membranes were then washed in 1×Tris-buffered saline, 0.1% Tween-20, pH 7.5 (TBST) and blocked with a buffer containing 1% BSA and 1% skimmed milk in TBST). This was followed by incubation with primary antibodies (Supplemental Table 2) overnight at +4 °C, three washes with TBST and incubation with horseradish peroxidase-conjugated secondary antibodies (Jackson Immunoresearch) for 2 hours at RT. Signals were detected using SuperSignal™ West Pico Chemiluminescent Substrate (Thermo Fisher Scientific) following the manufacturer's instructions and detecting the chemiluminescence with a Fujifilm LAS-3000 imaging system.

Proximity Ligation Assay

The proximity ligation assay (PLA) to study potential interactions between ColXVIII, EGFR and integrin α6 in cultured MDA-MB-231 and JIMT-1 cells was performed with the Duolink Detection Kit (#DUO92101, Sigma-Aldrich). The cells were seeded at a density of 1×10^4 per well in an 8-well Nunc Lab-Tek II Chamber Slide System (Thermo Fisher Scientific) and grown overnight. They were then briefly washed with PBS, followed by fixation in ice-cold 4% PFA for 10 min and permeabilized with 0.1% triton X-100 in PBS for 10 min. The cells were washed thrice for 5 min with PBS, after which the PLA protocol described by the manufacturer was followed (Duolink® PLA Fluorescence Protocol, Sigma-Aldrich). The primary antibodies used in the PLA assay were mouse monoclonal AB (mAB) DB144-N2 (1) for ColXVIII, dilution 1:150; rabbit mAB for EGFR (Abcam #52894), dilution 1:100; and rabbit polyclonal AB (pAB) for α6 integrin (Abcam, #97760), dilution 1:100 (Supplemental Table 2). The slides were mounted in Duolink® PLA Mounting Medium with DAPI, and the images were captured using a Zeiss LSM700 Microscope with a 100× oil immersion objective. Quantification of human ColXVIII, EGFR and ITGA6 protein-protein complexes was assessed by counting particles (red signals) using the ImageJ software. Minimum particle size was set to 5 pixels. Duolink signals were counted in 20

JIMT-1 and MDA-MB-231 cells for each interaction and presented as average with s.d. marked in whiskers.

Co-immunoprecipitation

Whole-cell lysates prepared in NETN-300 buffer from a sub-confluent 10 cm dish of MCF10A, SKBR3, JIMT1 and MDA-MB-231 cells were used for immunoprecipitation with antibodies against ColXVIII, EGFR and HER2, and integrins α6 and β1. For immune complex formation with the target molecule in the lysate, 5 μg of anti-EGFR (Abcam #52894), anti-ColXVIII (DB144-N2 or QH48.14)(1, 2), anti-α6 integrin AB (Abcam, #ab97760), or anti-β1-Integrin (Santa Cruz SC9970) antibody (Supplemental Table 2) was incubated with 1 ml of cell lysate overnight at +4°C with gentle rocking. The immune complex was then captured on Protein A- or G-coupled magnetic beads (Dynabeads, Invitrogen) and incubation was continued overnight at +4°C. The protein complex bound on the beads was pelleted with a magnetic separation rack and the beads were washed thrice with PBS. Immunoprecipitated proteins were eluted from the support in Laemlli loading buffer, separated by SDS-PAGE and detected by Western blotting using ColXVIII (QH48.14), EGFR (Cell Signaling Technology #4267), HER2 (Cell Signaling Technology #4290), integrin α6 (BD Biosiences #555734) and integrin β1 (Sigma Aldrich #AB1952) antibodies (Supplemental Table 2).

Fluorescence-activated cell sorting (FACS)

The single cell suspensions from human BC cell lines were prepared by trypsinization, after which the action of trypsin was blocked with complete medium containing FBS, centrifuged and resuspended in PBS for further analyses. Mouse mammary tumor tissue was extracted, minced and incubated in RPMI-1640 (Sigma-Aldrich) medium containing 0.3% collagenase, type 1 (CLS-1, Worthington) for 1 hour at 37°C, and filtered through a 70-µm mesh (BD Biosciences) to obtain single cell suspensions. The cell suspensions were then washed with PBS containing 4% FBS and taken for FACS analyses. All the samples prepared from mouse tissues or human cell lines were analysed on either LSR Fortessa or Aria II flow cytometers equipped with FACSDiva Software, or on a FACS Calibur cytometer equipped with CellQuest software (all from BD Biosciences). The following antibodies were used: fluorescent anti-CD24, -CD29, -CD44, -CD49f and Lineage antibody cocktail (CD3, CD11b, CD45R/B220, Ter-119, Ly-6G/C) (Supplemental Table 2). Enriched mammary SC or putative CSC populations from mouse mammary tumors were immunophenotyped as (Lin^{-}CD24^{+}CD44^{+}CD29^{High} CD49f^{High}) and those from human cell lines as (CD44^{+}CD24$^{-/low}$). All FACS data were analysed using Flowjo software (version 7.6.3, Tree Star Inc.).

ErbB inhibitor treatments in human breast cancer cell lines

Human BC that expressed high levels of HER2 receptor (SKBR-3, JIMT-1 and BT474) and triple negative MDA-MB-231 were used for the drug tests. Cells were seeded at a density of 1×10^4 cells per well in a 96-well format. For *COL18A1* gene expression knockdown, the two siRNAs that target *COL18A1* transcripts or scrambled siRNA as a negative control, were transfected into the cells as explained above. 48 hours post transfection the cells were washed with growth medium and EGFR/HER2 targeting drugs were added (lapatinib in DMSO at a final concentration of 2µM), trastuzumab (at 12.5µg/ml) or panitumumab (at 1.5µg/ml). The control scrambled and control ColXVIII KD cells received the vehicle only instead of the drugs. Cell proliferation and migration

were monitored for 30-120 h after drug treatment using the IncuCyte live cell analysis system as described above in connection with the cell proliferation and migration assays.

In vivo lapatinib treatment

WT-PyMT and $18^{-/-}$-PyMT mice were used for the *in vivo* lapatinib efficacy study. 7-weeks old female mice were treated with two different doses of lapatinib ditosylate (LC laboratories), either 35 or 70 mg per kilogram of body weight (mpk) twice a day and five days a week for a total of three weeks. For metastasis follow up experiment, a similar regimen with 70 mpk dose was used (Fig. 8D). The 70 mpk dose was chosen to be half the optimized human dose according published guidelines (18). Lapatinib was given as an oral suspension in a vehicle consisting of water with 0.5% Carboxymethylcellulose and 0.1% Tween-80. Vehicle-treated WT-PyMT and $18^{-/-}$-PyMT mice were included as controls. The animals were regularly monitored with respect to body weight and tumor development. The mammary glands were collected after three weeks of treatment, weighed at the time of sacrifice and samples were harvested for processing and further histological analyses. In metastasis experiment, the WT-PyMT mice were followed up to 14 weeks. The WT groups could not be further maintained because their primary tumor burden exceeded the humane end point. $18^{-/-}$-PyMT control and lapatinib 70 mpk groups were maintained for 17 weeks. At the end of the experiments, mice were sacrificed, primary tumors and lungs were harvested, and lungs were observed for gross pulmonary metastasis, and samples were processed for further histological analyses. Lung metastasis area was measured using Fiji ImageJ software.

REFERENCES CITED IN THE SUPPLEMENT

1. Valtola R et al. VEGFR-3 and its ligand VEGF-C are associated with angiogenesis in breast cancer. *Am. J. Pathol.* 1999;154(5):1381–1390.

2. Saarela J, Rehn M, Oikarinen A, Autio-Harmainen H, Pihlajaniemi T. The short and long forms of type XVIII collagen show clear tissue specificities in their expression and location in basement membrane zones in humans. *Am. J. Pathol.* 1998;153(2):611–626.

3. Aikio M et al. Specific collagen XVIII isoforms promote adipose tissue accrual via mechanisms determining adipocyte number and affect fat deposition. *Proc. Natl. Acad. Sci. U. S. A.* 2014;111(30):E3043–E3052.

4. Zaferani A et al. Basement membrane zone collagens XV and XVIII/proteoglycans mediate leukocyte influx in renal ischemia/reperfusion. *PLoS One* 2014;9(9):e106732.

5. Győrffy B. Survival analysis across the entire transcriptome identifies biomarkers with the highest prognostic power in breast cancer. *Comput. Struct. Biotechnol. J.* 2021;19:4101–4109.

6. Lian X et al. Defining the Extracellular Matrix of Rhabdomyosarcoma. *Front. Oncol.* 2021;11:601957.

7. Fluck MM, Schaffhausen BS. Lessons in Signaling and Tumorigenesis from Polyomavirus Middle T Antigen. *Microbiol. Mol. Biol. Rev.* 2009;73(3):542–563.

8. Dai X, Cheng H, Bai Z, Li J. Breast cancer cell line classification and Its relevance with breast tumor subtyping. *J. Cancer* 2017;8(16):3131–3141.

9. Rouillard AD et al. The harmonizome: a collection of processed datasets gathered to serve and mine knowledge about genes and proteins. *Database (Oxford).* 2016:baw100.

10. Tanner M et al. Characterization of a novel cell line established from a patient with Herceptin-resistant breast cancer. Mol. Cancer Ther. 2004; 3(12):1585–1592.

11. Neve RM et al. A collection of breast cancer cell lines for the study of functionally distinct cancer subtypes. *Cancer Cell* 2006;10(6):515–527.

12. Subik K et al. The expression patterns of ER, PR, HER2, CK5/6, EGFR, KI-67 and AR by immunohistochemical analysis in breast cancer cell lines. *Breast Cancer (Auckl)* 2010;4:35–41.

13. Rask G et al. Correlation of tumour subtype with long-term outcome in small breast carcinomas: a Swedish population-based retrospective cohort study. *Breast Cancer Res. Treat.* 2022;195(3):367–377.

14. Alahuhta I et al. Endostatin induces proliferation of oral carcinoma cells but its effect on invasion is modified by the tumor microenvironment. *Exp. Cell Res.* 2015;336(1):130–140.

15. Fukai N et al. Lack of collagen XVIII/endostatin results in eye abnormalities. *EMBO J.* 2002;21(7):1535–1544.

16. Guy CT, Cardiff RD, Muller WJ. Induction of mammary tumors by expression of polyomavirus middle T oncogene: a transgenic mouse model for metastatic disease. *Mol. Cell. Biol.* 1992;12(3):954–961.

17. Brisken C, Socolovsky M, Lodish HF, Weinberg R. The signaling domain of the erythropoietin receptor rescues prolactin receptor-mutant mammary epithelium. *Proc. Natl. Acad. Sci. U. S. A.* 2002;99(22):14241–14245.

18. Nair A, Jacob S. A simple practice guide for dose conversion between animals and human. *J. Basic Clin. Pharm.* 2016;7(2):27–31.
